# Quasi-invariant Gaussian measures for the cubic fourth order nonlinear Schrödinger equation

**DOI:** 10.1007/s00440-016-0748-7

**Published:** 2016-12-23

**Authors:** Tadahiro Oh, Nikolay Tzvetkov

**Affiliations:** 1 School of Mathematics, The University of Edinburgh, James Clerk Maxwell Building, The King’s Buildings, Peter Guthrie Tait Road, Edinburgh, EH9 3FD UK; 2 The Maxwell Institute for the Mathematical Sciences, James Clerk Maxwell Building, The King’s Buildings, Peter Guthrie Tait Road, Edinburgh, EH9 3FD UK; 3 Université de Cergy-Pontoise, 2, Av. Adolphe Chauvin, 95302 Cergy-Pontoise Cedex, France

**Keywords:** Fourth order nonlinear Schrödinger equation, Biharmonic nonlinear Schrödinger equation, Gaussian measure, Quasi-invariance, 35Q55

## Abstract

We consider the cubic fourth order nonlinear Schrödinger equation on the circle. In particular, we prove that the mean-zero Gaussian measures on Sobolev spaces $H^s({\mathbb {T}})$, $s > \frac{3}{4}$, are quasi-invariant under the flow.

## Introduction

### Background

In this paper, we continue the program set up by the second author [70] and study the transport property of Gaussian measures on Sobolev spaces under the dynamics of a certain Hamiltonian partial differential equation (PDE).

In probability theory, there is an extensive literature on the transport property of Gaussian measures under linear and nonlinear transformations. See, for example, [3, 6, 19, 24, 25, 45, 62]. Classically, Cameron–Martin [19] studied the transport property of Gaussian measures under a shift and established a dichotomy between absolute continuity and singularity of the transported measure. In the context of nonlinear transformations, the work in [45, 62] considers nonlinear transformations that are close to the identity, while the work in [24, 25] considers the transport property under the flow generated by (non-smooth) vector fields. In particular, in [25], the existence of quasi-invariant measures under the dynamics was established under an exponential integrability assumption of the divergence of the corresponding vector field. We also note a recent work [53] establishing absolute continuity of the Gaussian measure associated to the complex Brownian bridge on the circle under certain gauge transformations.

In the field of Hamiltonian PDEs, Gaussian measures naturally appear in the construction of invariant measures associated to conservation laws such as Gibbs measures. These invariant measures associated to conservation laws are typically constructed as weighted Gaussian measures. There has been a significant progress over the recent years in this subject. See [8–12, 14–16, 18, 27, 28, 30, 31, 48–50, 52, 55–57, 61, 63, 65, 67–69, 71, 72, 74, 75]. On the one hand, in the presence of such an invariant weighted Gaussian measure, one can study the transport property of a specific Gaussian measure, relying on the mutual absolute continuity of the invariant measure and the Gaussian measure. On the other hand, the invariant measures constructed in the forementioned work are mostly supported on rough functions with the exception of completely integrable Hamiltonian PDEs such as the cubic nonlinear Schrödinger equation (NLS), the KdV equation, and the Benjamin–Ono equation [29, 71, 72, 74, 75]. These completely integrable equations admit conservation laws at high regularities, allowing us to construct weighted Gaussian measures supported on smooth functions. In general, however, it is rare to have a conservation law at a high regularity and thus one needs an alternative method to study the transport property of Gaussian measures supported on smooth functions under the dynamics of non-integrable PDEs.

In the following, we consider the cubic fourth order NLS as a model equation and study the transport property of Gaussian measures supported on smooth functions. In particular, we prove that the transported Gaussian measures and the original Gaussian measures are mutually absolutely continuous with respect to each other. Our approach combines PDE techniques such as an energy estimate and normal form reductions and probabilistic techniques in an intricate manner.

### Cubic fourth order nonlinear Schrödinger equation

As a model dispersive equation, we consider the cubic fourth order nonlinear Schrödinger equation on ${\mathbb {T}}$:

1.1 $$\begin{aligned} {\left\{ \begin{array}{ll} i \partial _tu = \partial _x^4 u \pm |u|^{2}u \\ u|_{t = 0} = u_0, \end{array}\right. } \quad (x, t) \in {\mathbb {T}}\times {\mathbb {R}}, \end{aligned}$$

where *u* is a complex-valued function on ${\mathbb {T}}\times {\mathbb {R}}$ with ${\mathbb {T}}= {\mathbb {R}}/(2\pi {\mathbb {Z}})$. The Eq. (1.1) is also called the biharmonic NLS and it was studied in [40, 66] in the context of stability of solitons in magnetic materials. The biharmonic NLS (1.1) is a special case of the following more general class of fourth order NLS:

1.2 $$\begin{aligned} i \partial _tu = \lambda \partial _x^2 u + \mu \partial _x^4 u \pm |u|^{2}u. \end{aligned}$$

The model (1.2) was introduced in [41, 42] to include the effect of small fourth-order dispersion terms in the propagation of intense laser beams in a bulk medium with Kerr nonlinearity. See also [5, 33, 60] for the references therein.

The Eq. (1.1) is a Hamiltonian PDE with the following Hamiltonian:

1.3 $$\begin{aligned} H(u) = \frac{1}{2} \int _{\mathbb {T}}|\partial _x^2 u |^2 dx \pm \frac{1}{4}\int _{\mathbb {T}}|u|^4 dx. \end{aligned}$$

Moreover, the mass *M*(*u*) defined by

1.4 $$\begin{aligned} M(u) = \int _{\mathbb {T}}|u|^2 dx \end{aligned}$$

is conserved under the dynamics of (1.1). This mass conservation allows us to prove the following global well-posedness of (1.1) in $L^2({\mathbb {T}})$.

#### Proposition 1.1

The cubic fourth order NLS (1.1) is globally well-posed in $H^s({\mathbb {T}})$ for $s \ge 0$.

See Appendix A for the proof. We point out that Proposition 1.1 is sharp in the sense that (1.1) is ill-posed below $L^2({\mathbb {T}})$. See the discussion in Sect. A.2. See also [38, 59].

Our main goal is to study the transport property of Gaussian measures on Sobolev spaces under the dynamics of (1.1).

### Main result

We first introduce a family of mean-zero Gaussian measures on Sobolev spaces. Given $s> \frac{1}{2}$, let $\mu _s$ be the mean-zero Gaussian measure on $L^2({\mathbb {T}})$ with the covariance operator $2(\text {Id} - \Delta )^{-s}$, written as

1.5 $$\begin{aligned} d \mu _s = Z_s^{-1} e^{-\frac{1}{2} \Vert u\Vert _{H^s}^2} du = Z_s^{-1} \prod _{n \in {\mathbb {Z}}} e^{-\frac{1}{2} \langle n \rangle ^{2s} |\widehat{u}_n|^2} d\widehat{u}_n . \end{aligned}$$

While the expression $d \mu _s = Z_s^{-1} \exp (-\frac{1}{2} \Vert u\Vert _{H^s}^2 )du$ may suggest that $\mu _s$ is a Gaussian measure on $H^s({\mathbb {T}})$, we need to enlarge a space in order to make sense of $\mu _s$.

The Gaussian measure $\mu _s$ defined above is in fact the induced probability measure under the map^1^

1.6 $$\begin{aligned} \omega \in \Omega \mapsto u^\omega (x) = u(x; \omega ) = \sum _{n \in {\mathbb {Z}}} \frac{g_n(\omega )}{\langle n \rangle ^s}e^{inx}, \end{aligned}$$

where $\langle \,\cdot \, \rangle = (1+|\cdot |^2)^\frac{1}{2}$ and $\{ g_n \}_{n \in {\mathbb {Z}}}$ is a sequence of independent standard complex-valued Gaussian random variables, i.e. $\text {Var}(g_n) = 2$. Note that $u^\omega$ in (1.6) lies in $H^{\sigma }({\mathbb {T}})$ for $\sigma < s -\frac{1}{2}$ but not in $H^{s-\frac{1}{2}}({\mathbb {T}})$ almost surely. Moreover, for the same range of $\sigma$, $\mu _s$ is a Gaussian probability measure on $H^{\sigma }({\mathbb {T}})$ and the triplet $(H^s, H^\sigma , \mu _s)$ forms an abstract Wiener space. See [36, 46].

Recall the following definition of quasi-invariant measures. Given a measure space $(X, \mu )$, we say that $\mu$ is *quasi-invariant* under a transformation *T*: $X \rightarrow X$ if the transported measure $T_*\mu = \mu \circ T^{-1}$ and $\mu$ are equivalent, i.e. mutually absolutely continuous with respect to each other. We now state our main result.

#### Theorem 1.2

Let $s > \frac{3}{4}$. Then, the Gaussian measure $\mu _s$ is quasi-invariant under the flow of the cubic fourth order NLS (1.1).

When $s = 2$, one may obtain Theorem 1.2 by establishing invariance of the Gibbs measure “$d\rho = Z^{-1} \exp (-H(u))du$” and appealing to the mutual absolute continuity of the Gibbs measure $\rho$ and the Gaussian measure $\mu _2$, at least in the defocusing case. Such invariance, however, is a very rigid statement and is not applicable to other values of $s >\frac{3}{4}$.

Instead, we follow the approach introduced by the second author in the context of the (generalized) BBM equation [70]. In particular, we combine both PDE techniques and probabilistic techniques in an intricate manner. Moreover, we perform both local and global analysis on the phase space. An example of local analysis is an energy estimate (see Proposition 6.1 below), where we study a property of a particular trajectory, while examples of global analysis include the transport property of Gaussian measures under global transformations discussed in Sect. 4 and a change-of-variable formula (Proposition 6.6).

As in [70], it is essential to exhibit a smoothing on the nonlinear part of the dynamics of (1.1). Furthermore, we crucially exploit the invariance property of the Gaussian measure $\mu _s$ under some nonlinear (gauge) transformation. See Sect. 4. In the context of the generalized BBM considered in [70], there was an obvious smoothing coming from the smoothing operator applied to the nonlinearity. There is, however, no apparent smoothing for our Eq. (1.1). In fact, a major novelty compared to [70] is that in this work we exploit the dispersive nature of the equation in a fundamental manner. Our main tool in this context is normal form reductions analogous to the approach employed in [4, 37, 47]. In [4], Babin–Ilyin–Titi introduced a normal form approach for constructing solutions to dispersive PDEs. It turned out that this approach has various applications such as establishing unconditional uniqueness [37, 47] and exhibiting nonlinear smoothing [32]. The normal form approach is also effective in establishing a good energy estimate, though such an application of the normal form reduction in energy estimates is more classical and precedes the work of [4]. See Sect. 6.1.

In [62], Ramer proved a criterion on quasi-invariance of a Gaussian measure on an abstract Wiener space under a nonlinear transformation. In the context of our problem, this result basically states that $$\mu _s$$ is quasi-invariant if the nonlinear part is $$(1+\varepsilon )$$-smoother than the linear part. See [45] for a related previous result. In Sect. 5, we perform a normal form reduction on the renormalized Eq. (3.6) and exhibit $$(1+\varepsilon )$$-smoothing on the nonlinear part if $$s > 1$$. This argument provides the first proof of Theorem 1.2 when $$ s > 1$$. It seems that the regularity restriction $$s > 1$$ is optimal for the application of Ramer’s result. See Remark 5.4.

When $s \le 1$, we need to go beyond Ramer’s argument. In this case, we follow the basic methodology in [70], combining an energy estimate and global analysis of truncated measures. Due to a lack of apparent smoothing, our energy estimate is more intricate. Indeed, we need to perform a normal form reduction and introduce a modified energy for this purpose. This introduces a further modification to the argument from [70]. See Sect. 6. Lastly, let us point out the following. While the regularity restriction $s > \frac{3}{4}$ in Theorem 1.2 comes from the energy estimate (Proposition 6.1), we expect that, by introducing some new ideas related to more refined normal form reductions developed in [37], the result may be extended to the (optimal) regularity range $s>\frac{1}{2}$. We plan to address this question in a future work.

#### Remark 1.3

(i) In the higher regularity setting $s > 1$, we can reduce the proof of Theorem 1.2 to Ramer’s result [62]. See Sect. 5. While there is an explicit representation for the Radon–Nikodym derivative in [62], we do not know how to gain useful information from it at this point

(ii) In the low regularity case $\frac{3}{4} < s \le 1$, we employ the argument introduced in [70]. See Sect. 6. This argument is more quantitative and in particular, it allows us to obtain a polynomial upper bound on the growth of the Sobolev norm. However, such a polynomial growth bound may also be obtained by purely deterministic methods. See Remark 7.4 in [70]. A quasi-invariance result with better quantitative bounds may lead to an improvement of the known deterministic bounds. At the present moment, however, we do not know how to make such an idea work.

(iii) We point out that the existence of a quasi-invariant measure is a qualitative statement, showing a delicate persistency property of the dynamics. In particular, this persistence property due to the quasi-invariance is stronger than the (usual) persistence of regularity. In a future work, we plan to construct Hamiltonian dynamics possessing the persistence of regularity such that the Gaussian measure $\mu _s$ and the transported measure under the dynamics are mutually singular.

#### Remark 1.4

Let us briefly discuss the situation for the related cubic (second order) NLS:

1.7 $$\begin{aligned} i \partial _tu = \partial _x^2 u \pm |u|^{2}u, \quad (x, t) \in {\mathbb {T}}\times {\mathbb {R}}. \end{aligned}$$

It is known to be completely integrable and possesses an infinite sequence of conservation laws $H_k$, $k \in {\mathbb {N}}\cup \{0\}$, controlling the $H^k$-norm [1, 2, 35]. Associated to the conservation laws $H_k$, $k \ge 1$, there exists an infinite sequence of invariant weighted Gaussian measures $\rho _k$ supported on $H^{k - \frac{1}{2} -\varepsilon }({\mathbb {T}})$, $\varepsilon > 0$ [8, 74]. As mentioned above, one may combine this invariance and the mutual absolute continuity of $\rho _k$ and the Gaussian measure $\mu _k$ to deduce quasi-invariance of $\mu _k$ under the dynamics of (1.7), $k \ge 1$. It may be of interest to investigate quasi-invariance of $\mu _s$ for non-integer values of *s*.

### Organization of the paper

In Sect. 2, we introduce some notations. In Sect. 3, we apply several transformations to (1.1) and derive a new renormalized equation. We also prove a key factorization lemma (Lemma 3.1) which play a crucial role in the subsequent nonlinear analysis. We then investigate invariance properties of Gaussian measures under several transformations in Sect. 4. In Sect. 5, we prove Theorem 1.2 for $s > 1$ as a consequence of Ramer’s result [62]. By establishing a crucial energy estimate and performing global analysis of truncated measures, we finally present the proof of Theorem 1.2 for the full range $s > \frac{3}{4}$ in Sect. 6. In Appendix A, we discuss the well-posedness issue of the Cauchy problem (1.1). Then, we use it to study the approximation property of truncated dynamics in Appendix B, which is used in the proof of Theorem 1.2 in Sect. 6.

## Notations

Given $N \in {\mathbb {N}}$, we use ${\mathbf {P}}_{\le N}$ to denote the Dirichlet projection onto the frequencies $\{|n|\le N\}$ and set ${\mathbf {P}}_{> N} := \text {Id} - {\mathbf {P}}_{\le N}$. Define $E_N$ and $E_N^\perp$ by

$$\begin{aligned} E_N&= {\mathbf {P}}_{\le N} L^2({\mathbb {T}}) = \text {span}\{e^{inx}: |n|\le N\},\\ E_N^\perp&= {\mathbf {P}}_{>N} L^2({\mathbb {T}}) = \text {span}\{e^{inx}: |n|> N\}. \end{aligned}$$

Given $s> \frac{1}{2}$, let $\mu _s$ be the Gaussian measure on $L^2({\mathbb {T}})$ defined in (1.5). Then, we can write $\mu _s$ as

2.1 $$\begin{aligned} \mu _s = \mu _{s, N}\otimes \mu _{s, N}^\perp , \end{aligned}$$

where $\mu _{s, N}$ and $\mu _{s, N}^\perp$ are the marginal distributions of $\mu _s$ restricted onto $E_N$ and $E_N^\perp$, respectively. In other words, $\mu _{s, N}$ and $\mu _{s, N}^\perp$ are induced probability measures under the following maps:

2.2 $$\begin{aligned}&u_N :\omega \in \Omega \mapsto u_N (x; \omega ) = \sum _{|n|\le N} \frac{g_n(\omega )}{\langle n \rangle ^s}e^{inx}, \end{aligned}$$

2.3 $$\begin{aligned}&u_N^\perp : \omega \in \Omega \mapsto u_N^\perp (x; \omega ) = \sum _{|n|>N} \frac{g_n(\omega )}{\langle n \rangle ^s}e^{inx}, \end{aligned}$$

respectively. Formally, we can write $\mu _{s, N}$ and $\mu _{s, N}^\perp$ as

2.4 $$\begin{aligned} d \mu _{s, N} = Z_{s, N}^{-1} e^{-\frac{1}{2} \Vert {\mathbf {P}}_{\le N} u_N\Vert _{H^s}^2} d u_N \quad \text {and} \quad d \mu _{s, N}^\perp = \widehat{Z}_{s,N }^{-1} e^{-\frac{1}{2} \Vert {\mathbf {P}}_{>N} u_N^\perp \Vert _{H^s}^2} d u_N^\perp . \end{aligned}$$

Given $r > 0$, we also define a probability measure $\mu _{s, r}$ by

2.5 $$\begin{aligned} d \mu _{s, r} = Z_{s, r}^{-1}{\mathbf {1}}_{\{ \Vert v\Vert _{L^2 } \le r\}} d\mu _s. \end{aligned}$$

The defocusing/focusing nature of the Eq. (1.1) does not play any role, and thus we assume that it is defocusing, i.e. with the $+$ sign in (1.1). Moreover, in view of the time reversibility of the equation, we only consider positive times in the following.

## Reformulation of the cubic fourth order NLS

In this section, we apply several transformations to (1.1) and reduce it to a convenient form on which we perform our analysis. Given $t \in {\mathbb {R}}$, we define a gauge transformation ${\mathcal {G}}_t$ on $L^2({\mathbb {T}})$ by setting3.1where . Given a function $u \in C({\mathbb {R}}; L^2({\mathbb {T}}))$, we define ${\mathcal {G}}$ by setting

$$\begin{aligned} {\mathcal {G}}[u](t) : = {\mathcal {G}}_t[u(t)]. \end{aligned}$$

Note that ${\mathcal {G}}$ is invertible and its inverse is given by ${\mathcal {G}}^{-1}[u](t) = {\mathcal {G}}_{-t}[u(t)]$.

Let $u \in C({\mathbb {R}}; L^2({\mathbb {T}}))$ be a solution to (1.1). Define $\widetilde{u}$ by3.2Then, it follows from the the mass conservation that $\widetilde{u}$ is a solution to the following renormalized fourth order NLS:3.3Next, define the interaction representation *v* of $\widetilde{u}$ by

3.4 $$\begin{aligned} v(t) = S(-t)\widetilde{u}(t), \end{aligned}$$

where $S(t) = e^{-i t\partial _x^4}$. For simplicity of notations, we use $v_n$ to denote the Fourier coefficient of *v* in the following, when there is no confusion. By writing (3.4) on the Fourier side, we have

3.5 $$\begin{aligned} v_n(t) = e^{ it n^4} \widetilde{u}_n(t). \end{aligned}$$

Then, with (3.5), we can reduce (3.3) to the following equation for $\{v_n\}_{n \in {\mathbb {Z}}}$:

3.6 $$\begin{aligned} \partial _tv_n&= -i e^{i t n^4} ( i \partial _t\widetilde{u}_n - n^4 \widetilde{u}_n)\nonumber \\&= -i \sum _{\Gamma (n)} e^{-i \phi ({\bar{n}}) t} v_{n_1}\overline{v_{n_2}}v_{n_3} + i |v_n|^2 v_n \nonumber \\&=: {\mathcal {N}}(v)_n + {\mathcal {R}}(v)_n, \end{aligned}$$

where the phase function $\phi ({\bar{n}})$ and the plane $\Gamma (n)$ are given by

3.7 $$\begin{aligned} \phi ({\bar{n}}) = \phi (n_1, n_2, n_3, n) = n_1^4 - n_2^4 + n_3^4 - n^4 \end{aligned}$$

and

3.8 $$\begin{aligned} \Gamma (n) = \{(n_1, n_2, n_3) \in {\mathbb {Z}}^3:\, n = n_1 - n_2 + n_3 \text { and } n_1, n_3 \ne n\}. \end{aligned}$$

The phase function $\phi ({\bar{n}})$ admits the following factorization.

### Lemma 3.1

Let $n = n_1 - n_2 + n_3$. Then, we have

3.9 $$\begin{aligned} \phi ({\bar{n}}) = (n_1 - n_2)(n_1-n) \big ( n_1^2 +n_2^2 +n_3^2 +n^2 + 2(n_1 +n_3)^2\big ). \end{aligned}$$

### Proof

With $$n = n_1 - n_2 + n_3 $$, we have

3.10 $$\begin{aligned} \phi ({\bar{n}})&= (n_1 - n_2) \Big \{(n_1^3 + n_2^3 -n_3^3 - n^3 ) + ( n_1^2 n_2 + n_1 n_2^2 - n_3^2 n - n_3 n^2 )\Big \} \nonumber \\&= :(n_1 - n_2)( \text {I}+ \text {II}). \end{aligned}$$

On the one hand, we have

3.11 $$\begin{aligned} \text {I}= (n_1 - n) ( n_1^2 + n_1 n + n^2 + n_2^2 + n_2 n_3 + n_3^2 ). \end{aligned}$$

On the other hand, with $n_2 = n_1 + n_3 - n$, we have

3.12 $$\begin{aligned} \text {II}&= 2n_1^3 + 3 (n_3 - n) n_1^2 + (n_3^2 - 2n_3 n +n^2) n_1 - n_3^2n - n_3 n^2\nonumber \\&= (n_1 - n) \big ( 2n_1^2 + (3n_3 - n) n_1 + n_3^2 + n_3 n\big ). \end{aligned}$$

From (3.10) with (3.10) and (3.12) with $n_2 = n_1 + n_3 - n$, we obtain

$$\begin{aligned} \phi ({\bar{n}})&= (n_1 - n_2)(n_1 - n)(3n_1^2 + n_2^2 + 2n_3^2 + n^2 + 3 n_1 n_3 + n_2 n_3 + n_3 n)\\&= (n_1 - n_2)(n_1 - n)\big ( n_1^2 +n_2^2 +n_3^2 +n^2 + 2(n_1 +n_3)^2\big ). \end{aligned}$$

$$\square $$

In the remaining part of the paper, we present the proof of Theorem 1.2 by performing analysis on (3.6). In view of Lemma 3.1, we refer to the first term ${\mathcal {N}}(v)$ and the second term ${\mathcal {R}}(v)$ on the right-hand side of (3.6) as the non-resonant and resonant terms, respectively. While we do not have any smoothing on ${\mathcal {R}}(v)$ under a time integration, Lemma 3.1 shows that there is a smoothing on the non-resonant term ${\mathcal {N}}(v)$. We will exploit this fact in Sect. 5. In Sect. 6, we will exploit a similar non-resonant behavior in establishing a crucial energy estimate (Proposition 6.1).

## Gaussian measures under transformations

In this section, we discuss invariance properties of Gaussian measures under various transformations.

### Lemma 4.1

Let $t \in {\mathbb {R}}$. Then, the Gaussian measure $\mu _s$ defined in (1.5) is invariant under the linear map *S*(*t*).

### Proof

Note that $\mu _s$ can be written as an infinite product of Gaussian measures:

$$\begin{aligned} \mu _s = \bigotimes _{n \in {\mathbb {Z}}} \rho _n, \end{aligned}$$

where $\rho _n$ is the probability distribution for $\widehat{u}_n$. In particular, $\rho _n$ is a mean-zero Gaussian probability measure on ${\mathbb {C}}$ with variance $2 \langle n \rangle ^{-2s}$. Then, noting that the action of *S*(*t*) on $\widehat{u}_n$ is a rotation by $e^{-i tn^4}$, the lemma follows from the rotation invariance of each $\rho _n$. $\square$

### Lemma 4.2

Given a complex-valued mean-zero Gaussian random variable *g* with variance $$\sigma $$, i.e. $$g \in {\mathcal {N}}_{\mathbb {C}}(0, \sigma )$$, let $$ Tg = e^{i t |g|^2} g$$ for some $$t \in {\mathbb {R}}$$. Then, $$Tg \in {\mathcal {N}}_{\mathbb {C}}(0, \sigma )$$.

### Proof

By viewing ${\mathbb {C}}\simeq {\mathbb {R}}^2$, let $\mathbf{x} = (x, y ) = ({\hbox {Re }}g, {\hbox {Im }}g)$ and $\mathbf{u} = (u, v ) = ({\hbox {Re }}T g, {\hbox {Im }}T g)$. Noting that $|Tg| = |g|$, we have $T^{-1} g = e^{-it | g|^2} g$. In terms of $\mathbf{x}$ and $\mathbf{u}$, we have

$$\begin{aligned} \mathbf{x} = T^{-1} \mathbf{u} = ( u \cos t |\mathbf{u}|^2 + v \sin t |\mathbf{u}|^2, - u \sin t |\mathbf{u}|^2 + v \cos t |\mathbf{u}|^2). \end{aligned}$$

Then, with $C_t = \cos t|\mathbf{u}|^2$ and $S_t = \sin t|\mathbf{u}|^2$, a direct computation yields

$$\begin{aligned} \det D_\mathbf{u} T^{-1}&= \left| \begin{matrix} C_t - 2t u^2 S_t + 2t uv C_t &{} S_t -2tuv S_t + 2t v^2 C_t\\ -S_t - 2t u^2 C_t - 2tuv S_t&{} C_t - 2t uv C_t - 2tv^2 S_t \end{matrix}\right| \\&= \big \{C_t^2 - 2 t uv C_t^2 - 2t v^2 S_t C_t\\&\quad - 2t u^2 S_t C_t + 4t^2 u^3 v S_t C_t + 4t^2 u^2 v^2 S_t^2\\&\quad + 2 t uv C_t^2 - 4 t^2 u^2 v^2 C_t^2 - 4 t^2 u v^3 S_t C_t\big \}\\&\quad - \big \{-S_t^2 + 2t uv S_t^2 - 2t v^2 S_t C_t\\&\quad -2 t u^2 S_t C_t + 4t^2 u^3 v S_t C_t - 4 t^2 u^2 v^2 C_t^2\\&\quad - 2t u v S_t^2 + 4 t^2 u^2 v^2 S_t^2 - 4 t^2 u v^3 S_t C_t\big \} \\&= 1. \end{aligned}$$

Let $\mu$ and $\widetilde{\mu }$ be the probability distributions for *g* and *Tg*. Then, for a measurable set $A \subset {\mathbb {C}}\simeq {\mathbb {R}}^2$, we have

$$\begin{aligned} \widetilde{\mu }(A)&= \mu (T^{-1}A) = \frac{1}{\pi \sigma }\int _{T^{-1}A} e^{-\frac{|\mathbf{x}|^2}{\sigma }} dx dy = \frac{1}{\pi \sigma }\int _{A} e^{-\frac{|T^{-1} \mathbf{u}|^2}{\sigma }} |\det D_\mathbf{u} T^{-1}| du dv\\&= \frac{1}{\pi \sigma }\int _{A} e^{-\frac{| \mathbf{u}|^2}{\sigma }} du dv = \mu (A). \end{aligned}$$

This proves the lemma. $\square$

Next, we extend Lemma 4.2 to the higher dimensional setting.

### Lemma 4.3

Let $s\in {\mathbb {R}}$ and $N \in {\mathbb {N}}$. Then, for any $t \in {\mathbb {R}}$, the Gaussian measure $\mu _{s, N}$ defined in (2.4) is invariant under the map ${\mathcal {G}}_t$ defined in (3.1).

While we could adapt the proof of Lemma 4.2 to the higher dimensional setting, this would involve computing determinants of larger and larger matrices. Hence, we present an alternative proof in the following.

### Proof

Given $$N \in {\mathbb {N}}$$, let $$E_N = \text {span}\{ e^{inx}: |n| \le N\}$$. Given $$u \in E_N$$, let $$v(t) = {\mathcal {G}}_t[u]$$ for $$t \in {\mathbb {R}}$$. Then, noting that $$\partial _tM(v(t)) = 0$$, where $$M(v(t)) = \sum _{|n|\le N} | v_n(t)|^2$$, we see that $$ v_n$$ satisfies the following system of ODEs:

4.1 $$\begin{aligned} d v_n = 2 i M(v) v_n dt, \quad |n|\le N, \end{aligned}$$

With $a_n = {\hbox {Re }}v_n$ and $b_n = {\hbox {Im }}v_n$, we can rewrite (4.1) as

4.2 $$\begin{aligned} {\left\{ \begin{array}{ll} d a_n = - 2 M(v) b_n dt\\ d b_n = 2 M(v) a_n dt, \end{array}\right. } \quad |n| \le N. \end{aligned}$$

Let ${\mathcal {L}}_N$ be the infinitesimal generator for (4.2). Then, $\mu _{s, N}$ is invariant under ${\mathcal {G}}_t$ for any $t \in {\mathbb {R}}$ if and only if $({\mathcal {L}}_N)^*\mu _{s, N} = 0$. See [44]. Note that the last condition is equivalent to

4.3 $$\begin{aligned} \int _{(a, b) \in {\mathbb {R}}^{2N+2}} {\mathcal {L}}_N F (a, b) d\mu _{s, N}(a, b) = 0 \end{aligned}$$

for all test functions $F\in C^\infty ({\mathbb {R}}^{2N+2}; {\mathbb {R}})$. From (4.2), we have

$$\begin{aligned} {\mathcal {L}}_N F(a, b) = \sum _{|n|\le N} \bigg ( - 2M(a, b) b_n \frac{\partial }{\partial a_n} +2 M(a, b) a_n \frac{\partial }{\partial b_n}\bigg ) F(a, b), \end{aligned}$$

where $M(a, b) = \sum _{|n|\le N} (a_n^2 + b_n^2)$. Then, by integration by parts, we have

$$\begin{aligned} \int _{(a, b) \in {\mathbb {R}}^{2N+2}}&{\mathcal {L}}_N F (a, b) d\mu _{s, N}(a, b) \nonumber \\&= 2 Z_N^{-1} \sum _{|n|\le N} \int _{ {\mathbb {R}}^{2N+2}} F(a, b) \frac{\partial }{\partial a_n}\bigg \{M(a, b) b_n e^{-\frac{1}{2} \sum _{|k|\le N} \frac{a_k^2}{\langle k \rangle ^{2s}} + \frac{b_k^2}{\langle k \rangle ^{2s}}} \bigg \} d a db \nonumber \\&\quad - 2 Z_N^{-1} \sum _{|n|\le N} \int _{ {\mathbb {R}}^{2N+2}} F(a, b) \frac{\partial }{\partial b_n}\bigg \{M(a, b) a_n e^{-\frac{1}{2} \sum _{|k|\le N} \frac{a_k^2}{\langle k \rangle ^{2s}} + \frac{b_k^2}{\langle k \rangle ^{2s}}} \bigg \} d a db\nonumber \\&= 4 Z_N^{-1} \sum _{|n|\le N} \int _{ {\mathbb {R}}^{2N+2}} F(a, b) \bigg ( 1 - \frac{M(a, b)}{2\langle n \rangle ^{2s}}\bigg ) a_n b_n e^{-\frac{1}{2} \sum _{|k|\le N} \frac{a_k^2}{\langle k \rangle ^{2s}} + \frac{b_k^2}{\langle k \rangle ^{2s}}} d a db \nonumber \\&\quad - 4 Z_N^{-1} \sum _{|n|\le N} \int _{ {\mathbb {R}}^{2N+2}} F(a, b) \bigg ( 1 - \frac{M(a, b)}{2\langle n \rangle ^{2s}}\bigg ) a_n b_n e^{-\frac{1}{2} \sum _{|k|\le N} \frac{a_k^2}{\langle k \rangle ^{2s}} + \frac{b_k^2}{\langle k \rangle ^{2s}}} d a db \\&= 0 \end{aligned}$$

This proves (4.3). $$\square $$

In the following, we assume that $s> \frac{1}{2}$ such that $\mu _s$ is a well-defined probability measure on $L^2({\mathbb {T}})$ and ${\mathcal {G}}_t$ defined in (3.1) makes sense on ${{\mathrm{supp}}}(\mu _s) = L^2({\mathbb {T}})$.

### Lemma 4.4

Let $s>\frac{1}{2}$. Then, for any $t \in {\mathbb {R}}$, the Gaussian measure $\mu _s$ defined in (1.5) is invariant under the map ${\mathcal {G}}_t$.

Note that, when $s = 1$, Lemma 4.4 basically follows from Theorem 3.1 in [53] which exploits the properties of the Brownian loop under conformal mappings. For general $s > \frac{1}{2}$, such approach does not seem to be appropriate. In the following, we present the proof, using Lemma 4.3.

### Proof

Fix $t \in {\mathbb {R}}$. Given $N \in {\mathbb {N}}$, let $F_N \in C_b(L^2({\mathbb {T}}); {\mathbb {R}})$ be a test function depending only on the frequencies $\{|n|\le N\}$. Then, we claim that

4.4 $$\begin{aligned} \int _{L^2} F_N\circ {\mathcal {G}}_t (u) d \mu _s (u) = \int _{L^2} F_N (u) d \mu _s(u). \end{aligned}$$

With a slight abuse of notations, we write

4.5 $$\begin{aligned} F_N(u) = F\big (\{u_n\}_{|n|\le N}\big ) = F_N(u_{-N}, u_{-N+1},\ldots , u_{ N-1}, u_N). \end{aligned}$$

Let $v = {\mathcal {G}}_t[u]$, where *u* is as in (1.6). Then, we have

$$\begin{aligned} v_n = e^{2 i t \sum _{k \in {\mathbb {Z}}} \frac{|g_k|^2}{\langle k \rangle ^{2s}}} \frac{g_n}{\langle n \rangle ^s} = e^{2 i t \sum _{|k| >N} \frac{|g_k|^2}{\langle k \rangle ^{2s}}} \cdot e^{2 i t \sum _{|k| \le N} \frac{|g_k|^2}{\langle k \rangle ^{2s}}}\frac{g_n}{\langle n \rangle ^s}. \end{aligned}$$

By the independence of $\{g_n \}_{|n|\le N}$ and $\{g_n \}_{|n|> N}$, we can write $\Omega = \Omega _0 \times \Omega _1$ such that

$$\begin{aligned} g_n (\omega ) = {\left\{ \begin{array}{ll} g_n(\omega _0), \ \omega _0 \in \Omega _0, &{} \text {if }|n|\le N, \\ g_n(\omega _1), \ \omega _1 \in \Omega _1, &{} \text {if }|n|> N. \end{array}\right. } \end{aligned}$$

Then, we have

4.6 $$\begin{aligned} \int _{L^2} F_N\circ {\mathcal {G}}_t (u) d \mu _s (u) = \int _{\Omega _1} I_N (\omega _1) d P(\omega _1), \end{aligned}$$

where $I_N(\omega _1)$ is given by

4.7 $$\begin{aligned} I_N(\omega _1) = \int _{\Omega _0} F_N\bigg ( \bigg \{ e^{2 i t \sum _{|k| >N} \frac{|g_k(\omega _1)|^2}{\langle k \rangle ^{2s}}}\cdot e^{2 i t \sum _{|k| \le N} \frac{|g_k(\omega _0)|^2}{\langle k \rangle ^{2s}}}\frac{g_n(\omega _0)}{\langle n \rangle ^s}\bigg \}_{|n|\le N}\bigg ) d P(\omega _0). \end{aligned}$$

Since $s > \frac{1}{2}$, we have $\mu (\omega _1) : = \sum _{|k| >N} \frac{|g_k(\omega _1)|^2}{\langle k \rangle ^{2s}} < \infty$ almost surely. For fixed $\omega _1 \in \Omega _1$, define $\{\widetilde{g}_n^{\omega _1}\}_{|n|\le N}$ by setting $\widetilde{g}_n^{\omega _1} = e^{2it \mu (\omega _1)} g_n$, $|n| \le N$. Then, by the rotational invariance of the standard complex-valued Gaussian random variables and independence of $\{g_n \}_{|n|\le N}$ and $\{g_n \}_{|n|> N}$, we see that, for almost every $\omega _1 \in \Omega _1$, $\{\widetilde{g}_n^{\omega _1}\}_{|n|\le N}$ is a sequence of independent standard complex-valued Gaussian random variables (in $\omega _0 \in \Omega _0$). In particular, the law of $\{\widetilde{g}_n^{\omega _1}\}_{|n|\le N}$ is the same as that of $\{ g_n\}_{|n|\le N}$, almost surely in $\omega _1 \in \Omega _1$. Then, from the definitions of $\mu _{s, N}$ and ${\mathcal {G}}_t$, we can rewrite (4.7) as

$$\begin{aligned} I_N(\omega _1)&= \int _{\Omega _0} F_N\bigg ( \bigg \{ e^{2 i t \sum _{|k| \le N} \frac{|\widetilde{g}_k^{\omega _1}(\omega _0)|^2}{\langle k \rangle ^{2s}}}\frac{\widetilde{g}_n^{\omega _1}(\omega _0)}{\langle n \rangle ^s}\bigg \}_{|n|\le N}\bigg ) d P(\omega _0)\\&= \int _{\Omega _0} F_N\bigg ( \bigg \{ e^{2 i t \sum _{|k| \le N} \frac{| g_k(\omega _0)|^2}{\langle k \rangle ^{2s}}}\frac{ g_n(\omega _0)}{\langle n \rangle ^s}\bigg \}_{|n|\le N}\bigg ) d P(\omega _0)\\&= \int _{E_N} F_N({\mathcal {G}}_t u_N) d\mu _{s, N}(u_N) \end{aligned}$$

for almost every $\omega _1 \in \Omega _1$, where $u_N = {\mathbf {P}}_{\le N} u$ is as in (2.2). Then, it follows from Lemma 4.3 with (4.5) and (2.1) that

4.8 $$\begin{aligned} I_N(\omega _1) = \int _{E_N} F_N({\mathcal {G}}_t u_N) d\mu _{s, N}(u_N) = \int _{E_N} F_N(u_N) d\mu _{s, N}(u_N) = \int _{L^2} F_N(u) d\mu _{s}(u), \end{aligned}$$

for almost every $\omega _1 \in \Omega _1$. Note that the right-hand side of (4.8) is independent of $\omega _1 \in \Omega _1$. Therefore, from (4.6) and (4.8), we have

$$\begin{aligned} \int _{L^2} F_N\circ {\mathcal {G}}_t (u) d \mu _s (u) = \int _{\Omega _1} \int _{L^2} F_N(u) d\mu _{s}(u) d P(\omega _1) = \int _{L^2} F_N(u) d\mu _{s}(u) \end{aligned}$$

This proves (4.4).

Next, given $F \in C_b(L^2({\mathbb {T}}); {\mathbb {R}})$, let $F_N(u) = F({\mathbf {P}}_{\le N} u)$, $N \in {\mathbb {N}}$. Then, $F_N(u)$ converges to *F*(*u*) almost surely with respect to $\mu _s$. Also, $F_N({\mathcal {G}}_t u)$ converges to $F({\mathcal {G}}_t u)$ almost surely with respect to $\mu _s$. Then, from the dominated convergence theorem and (4.4), we have

$$\begin{aligned} \int _{L^2} F\circ {\mathcal {G}}_t (u) d \mu _s (u)&= \lim _{N \rightarrow \infty } \int _{L^2} F_N\circ {\mathcal {G}}_t (u) d \mu _s (u) =\lim _{N\rightarrow \infty } \int _{L^2} F_N (u) d \mu _s(u)\\&= \int _{L^2} F (u) d \mu _s(u) \end{aligned}$$

for all $F \in C_b(L^2({\mathbb {T}}); {\mathbb {R}})$. Hence, the lemma follows (see, for example, [26, Proposition 1.5]). $\square$

Lastly, we conclude this section by stating the invariance property of quasi-invariance under a composition of two maps.

### Lemma 4.5

Let $(X, \mu )$ be a measure space. Suppose that $T_1$ and $T_2$ are maps on *X* into itself such that $\mu$ is quasi-invariant under $T_j$ for each $j = 1, 2$. Then, $\mu$ is quasi-invariant under $T = T_1 \circ T_2$.

### Proof

Suppose that $A \subset X$ is a measurable set such that $\mu (A) = 0$. By the quasi-invariance of $\mu$ under $T_1$, this is equivalent to $\mu (T_1^{-1}A) = 0$. Then, by the quasi-invariance of $\mu$ under $T_2$, the pushforward measure $T_*\mu$ satisfies

$$\begin{aligned} T_*\mu (A) = \mu (T^{-1}A) = \mu \big (T_2^{-1}(T_1^{-1}A)\big ) = 0. \end{aligned}$$

Conversely, if $T_*\mu (A) = 0$, then we have $\mu (T_1^{-1}A)=0$, which in turn implies $\mu (A) = 0$. Hence, $\mu$ and $T_*\mu$ are mutually absolutely continuous. $\square$

## Ramer’s argument: $s > 1$

In this section, we present the proof of Theorem 1.2 for $s > 1$. Our basic approach is to apply Ramer’s result after exhibiting a sufficient smoothing on the nonlinear part. As it is written, the Eq. (1.1) or (3.6) does not manifest a smoothing in an explicit manner. In the following, we perform a normal form reduction and establish a nonlinear smoothing by exploiting the dispersion of the equation.

### Normal form reduction

By writing (3.6) in the integral form, we have

5.1 $$\begin{aligned} v_n (t)&= v_n(0) -i \int _0^t \sum _{\Gamma (n)} e^{-i \phi ({\bar{n}}) t'} v_{n_1}\overline{v_{n_2}}v_{n_3}(t') dt' +i \int _0^t | v_{n}|^2v_{n}(t') dt'\nonumber \\&= : v_n(0) +{\mathfrak {N}}(v)(n, t)+{\mathfrak {R}}(v)(n, t). \end{aligned}$$

Lemma 3.1 states that we have a non-trivial (in fact, fast) oscillation caused by the phase function $\phi ({\bar{n}})$ in the non-resonant part ${\mathfrak {N}}(v)$. The main idea of a normal form reduction is to transform the non-resonant part ${\mathfrak {N}}(v)$ into smoother terms of higher degrees, exploiting this rapid oscillation. More concretely, integrating by parts, we formally have

5.2 $$\begin{aligned} {\mathfrak {N}}(v)(n, t)&= \sum _{\Gamma ( n)} \frac{ e^{-i \phi ({\bar{n}}) t'} }{\phi ({\bar{n}})} v_{n_1}(t')\overline{v_{n_2}(t')}v_{n_3}(t')\bigg |_{t' = 0}^t - \sum _{\Gamma ( n)} \int _0^t \frac{ e^{-i \phi ({\bar{n}}) t'} }{\phi ({\bar{n}})} \partial _t( v_{n_1}\overline{v_{n_2}}v_{n_3})(t') dt' \nonumber \\&= \sum _{\Gamma (n)} \frac{ e^{-i \phi ( {\bar{n}}) t} }{\phi ({\bar{n}})} v_{n_1}(t)\overline{v_{n_2}(t)}v_{n_3}(t) - \sum _{\Gamma ( n)} \frac{ 1}{\phi ({\bar{n}})} v_{n_1}(0)\overline{v_{n_2}(0)}v_{n_3}(0) \nonumber \\&\quad -2 \int _0^t \sum _{\Gamma (n)} \frac{ e^{-i \phi (\bar{n})t' } }{\phi ({\bar{n}})} \big \{ {\mathcal {N}}(v)_{n_1} + {\mathcal {R}}(v)_{n_1}\big \}\overline{v_{n_2}}v_{n_3}(t') dt' \nonumber \\&\quad - \int _0^t \sum _{\Gamma ( n)} \frac{ e^{-i \phi ({\bar{n}}) t' } }{\phi ({\bar{n}})} v_{n_1}\overline{\big \{ {\mathcal {N}}(v)_{n_2} + {\mathcal {R}}(v)_{n_2}\big \}}v_{n_3}(t') dt'\nonumber \\&=: \text {I}+ \text {II}+ \text {III}+ \text {IV}. \end{aligned}$$

In view of Lemma 3.1, the phase function $\phi ({\bar{n}})$ appearing in the denominators allows us to exhibit a smoothing in ${\mathfrak {N}}(v)$. See Lemma 5.1 below.

At this point, the computation in (5.2) is rather formal and thus requires justification in several steps. In the first step, we switched the order of the time integration and the summation:

5.3 $$\begin{aligned} -i \int _0^t \sum _{\Gamma (n)} e^{-i \phi ({\bar{n}}) t'} v_{n_1}\overline{v_{n_2}}v_{n_3}(t') dt' = -i \sum _{\Gamma (n)} \int _0^t e^{-i \phi ({\bar{n}}) t'} v_{n_1}\overline{v_{n_2}}v_{n_3}(t') dt'. \end{aligned}$$

With $w = {\mathcal {F}}^{-1}\big (|\widehat{v}_n|\big ) = \sum _{ n \in {\mathbb {Z}}} |\widehat{v}_n| e^{inx}$, we have

5.4 $$\begin{aligned} \sum _{\Gamma (n)} | v_{n_1}\overline{v_{n_2}}v_{n_3} | \le \Vert w \Vert _{L^3}^3 \lesssim \Vert w\Vert _{H^{\frac{1}{6}}}^3 = \Vert v\Vert _{H^{\frac{1}{6}}}^3. \end{aligned}$$

Hence, the sum $\sum _{\Gamma (n)} e^{-i \phi ({\bar{n}}) t'} v_{n_1}\overline{v_{n_2}}v_{n_3}(t')$ is absolutely convergent with a bound uniform in time $t'$, provided that $v \in C({\mathbb {R}}; H^s({\mathbb {T}}))$ with $s \ge \frac{1}{6}$. This justifies (5.3).

If $v \in C({\mathbb {R}}; H^s({\mathbb {T}}))$ with $s \ge \frac{1}{6}$, it follows from (3.6) and a computation similar to (5.4) that $v_n \in C^1({\mathbb {R}})$. This allows us to apply integration by parts and the product rule. Lastly, we need to justify the switching of the time integration and the summation in the last equality of (5.2). By crudely estimating with (3.6), (5.4) and Lemma 3.1 (note that $|\phi ({\bar{n}})| \ge 1$ on $\Gamma (n)$), we have

5.5 $$\begin{aligned} \sum _{\Gamma (n)} \bigg |\frac{ e^{-i \phi ({\bar{n}})t' } }{\phi ({\bar{n}})} \big \{ {\mathcal {N}}(v)_{n_1} + {\mathcal {R}}(v)_{n_1}\big \}\overline{v_{n_2}}v_{n_3}(t')\bigg |&\lesssim \Vert {\mathcal {N}}(v)_{n_1} + {\mathcal {R}}(v)_{n_1}\Vert _{\ell ^\infty _{n_1}} \sum _{n_2, n_3}\frac{|v_{n_2}v_{n_3}|}{\langle n_2 \rangle \langle n_3 \rangle } \nonumber \\&\lesssim \Vert v(t')\Vert _{H^{\frac{1}{6}}}^3 \Vert v(t')\Vert _{L^2}^2. \end{aligned}$$

Hence, the series on the left-hand side of (5.5) is absolutely convergent with a bound uniform in time $t'$, provided that $v \in C({\mathbb {R}}; H^s({\mathbb {T}}))$ with $s \ge \frac{1}{6}$. This justifies the last equality in (5.2).

The following lemma shows a nonlinear smoothing for (3.6). Note that the amount of smoothing for ${\mathfrak {R}}(v)$ depends on the regularity $s > \frac{1}{2}$.

#### Lemma 5.1

Let $s> \frac{1}{2}$. Then, we have

5.6 $$\begin{aligned} \Vert {\mathfrak {N}}(v)(t) \Vert _{H^{s+2}}&\lesssim \Vert v(0)\Vert _{H^s}^3 + \Vert v (t)\Vert _{H^s}^3 + t \sup _{t' \in [0, t]} \Vert v (t')\Vert _{H^s}^5, \end{aligned}$$

5.7 $$\begin{aligned} \Vert {\mathfrak {R}}(v)(t) \Vert _{H^{3s}}&\lesssim t \sup _{t' \in [0, t]} \Vert v (t')\Vert _{H^s}^3. \end{aligned}$$

#### Proof

By Lemma 3.1 and the algebra property of $H^s({\mathbb {T}})$, $s > \frac{1}{2}$, we have

$$\begin{aligned} \Vert \text {I}\Vert _{H^{s+2}}&\lesssim \bigg \Vert \langle n \rangle ^{s} \sum _{\Gamma (n)} |v_{n_1}(t)\overline{v_{n_2}(t)}v_{n_3}(t)| \bigg \Vert _{\ell ^2_n} \lesssim \bigg \Vert \langle n \rangle ^{s} \sum _{n = n_1 - n_2 + n_3} \prod _{j = 1}^3 |v_{n_j}(t)| \bigg \Vert _{\ell ^2_n} \\&= \big \Vert \{{\mathcal {F}}^{-1}(|\widehat{v}_n|)(t)\}^3\big \Vert _{H^s} \lesssim \Vert v(t)\Vert _{H^s}^3. \end{aligned}$$

The second term $\text {II}$ in (5.2) can be estimated in an analogous manner. Similarly, by Lemma 3.1, (3.6), and the algebra property of $H^s({\mathbb {T}})$, $s > \frac{1}{2}$, we have

$$\begin{aligned} \Vert \text {III}\Vert _{H^{s+2}}&\lesssim t \sup _{t'\in [0, t]} \bigg \Vert \langle n \rangle ^s \sum _{\Gamma (n)} \big |\big \{ {\mathcal {N}}(v)_{n_1} + {\mathcal {R}}(v)_{n_1}\big \}\overline{v_{n_2}}v_{n_3}(t')\big |\bigg \Vert _{\ell ^2_n}\\&= t \sup _{t'\in [0, t]} \big \Vert \{{\mathcal {F}}^{-1}(|\widehat{v}_n|)(t')\}^5\big \Vert _{H^s} \lesssim t \sup _{t'\in [0, t]} \Vert v(t')\Vert _{H^s}^5. \end{aligned}$$

The fourth term $\text {IV}$ in (5.2) can be estimated in an analogous manner. From (5.1) and $\ell ^2_n \subset \ell ^6_n$, we have

$$\begin{aligned} \Vert {\mathfrak {R}}(v)(t)\Vert _{H^{3s}}&\le t \sup _{t'\in [0, t]} \bigg (\sum _{n \in {\mathbb {Z}}} \langle n \rangle ^{6s} | v_{n}(t')|^6\bigg )^\frac{1}{2} = t \sup _{t'\in [0, t]} \Vert \langle n \rangle ^s v_n(t')\Vert _{\ell ^6_n}^3\\&\le t \sup _{t'\in [0, t]} \Vert \langle n \rangle ^s v_n(t')\Vert _{\ell ^2_n}^3 = t \sup _{t'\in [0, t]} \Vert v(t')\Vert _{H^s}^3. \end{aligned}$$

This proves the second estimate (5.7). $\square$

### Consequence of Ramer’s result

In this subsection, we present the proof of Theorem 1.2 for $s > 1$. The main ingredient is Ramer’s result [62] along with the nonlinear smoothing discussed in the previous subsection. We first recall the precise statement of the main result in [62] for readers’ convenience.

#### Proposition 5.2

(Ramer [62]) Let (*i*, *H*, *E*) be an abstract Wiener space and $\mu$ be the standard Gaussian measure on *E*. Suppose that $T = Id + K: U \rightarrow E$ be a continuous (nonlinear) transformation from some open subset $U\subset E$ into *E* such that

(i) *T* is a homeomorphism of *U* onto an open subset of *E*.
(ii) We have $K(U) \subset H$ and $K:U \rightarrow H$ is continuous.
(iii) For each $x \in U$, the map *DK*(*x*) is a Hilbert–Schmidt operator on *H*. Moreover, *DK*: $x \in U \rightarrow DK(x) \in HS(H)$ is continuous.
(iv) $Id _H + DK(x) \in GL(H)$ for each $x \in U$.

Then, $\mu$ and $\mu \circ T$ are mutually absolutely continuous measures on *U*.

Here, *HS*(*H*) denotes the space of Hilbert–Schmidt operators on *H* and *GL*(*H*) denotes invertible linear operators on *H* with a bounded inverse.

Given $t, \tau \in {\mathbb {R}}$, let $\Phi (t)$: $L^2\rightarrow L^2$ be the solution map for (1.1) and $\Psi (t, \tau )$: $L^2\rightarrow L^2$ be the solution map for (3.6),^2^ sending initial data at time $\tau$ to solutions at time *t*. When $\tau =0$, we may denote $\Psi (t, 0)$ by $\Psi (t)$ for simplicity.

By inverting the transformations (3.2) and (3.4) with (5.1), we have

5.8 $$\begin{aligned} \Phi (t)(u_0) = {\mathcal {G}}^{-1} \circ S(t) \circ \Psi (t)(u_0) = {\mathcal {G}}^{-1} \circ S(t) (u_0 + {\mathfrak {N}}(v)(t)+{\mathfrak {R}}(v)(t)), \end{aligned}$$

where $v(t) = \Psi (t)(u_0)$ and ${\mathfrak {N}}$ is given by (5.2). Now, write $\Psi (t) = \text {Id} + K(t)$, where

$$\begin{aligned} K(t)(u_0) : = {\mathfrak {N}}(v)(t)+{\mathfrak {R}}(v)(t) \end{aligned}$$

and *v* is the solution to (3.6) with $v|_{t = 0} = u_0$. In view of Lemmas 4.1, 4.4, and 4.5, it suffices to show that $\mu _s$ is quasi-invariant under $\Psi (t)$.

Fix $s > 1$ and $\sigma _1 > \frac{1}{2}$ sufficiently close to $\frac{1}{2}$. First, note that $\mu _s$ is a probability measure on $H^{s-\sigma _1}({\mathbb {T}})$. Given $R>0$, let $B_R$ be the open ball of radius *R* centered at the origin in $H^{s - \sigma _1}({\mathbb {T}})$. The following proposition shows that the hypotheses of Ramer’s result in [62] are indeed satisfied.

#### Proposition 5.3

Let $s > 1$. Given $R > 0$, there exists $\tau = \tau (R) > 0$ such that, for each $t \in (0, \tau (R)]$, the following statements hold:

(i) $\Psi (t)$ is a homeomorphism of $B_R$ onto an open subset of $H^{s-\sigma _1}({\mathbb {T}})$.
(ii) We have $K(t) (B_R) \subset H^s({\mathbb {T}})$ and $K(t): B_R \rightarrow H^s({\mathbb {T}})$ is continuous.
(iii) For each $u_0 \in B_R$, the map $DK(t)|_{u_0}$ is a Hilbert–Schmidt operator on $H^s({\mathbb {T}})$. Moreover, $DK(t): u_0 \in B_R \mapsto DK(t)|_{u_0} \in HS(H^s({\mathbb {T}}))$ is continuous.
(iv) $Id _{H^s} + DK(t)|_{u_0} \in GL (H^s({\mathbb {T}}))$ for each $u_0 \in B_R$.

We first present the proof of Theorem 1.2 for $s > 1$, assuming Proposition 5.3. Thanks to Ramer’s result (Proposition 5.2 above), Proposition 5.3 implies that $\mu _s$ and the pullback measure $\Psi (t)^*\mu _s := \mu _s\circ \Psi (t)$ are mutually absolutely continuous as measures restricted to the ball $B_R$ for any $t \in (0, \tau (R)]$.

*Proof of Theorem* 1.2 *for* $s > 1$ Given $R>0$, let $B_R \subset H^{s - \sigma _1}$ be the open ball of radius *R* centered at the origin as above. Fix $T > 0$. It follows from the growth estimate (A.12) of the $H^{s-\sigma _1}$-norm that

5.9 $$\begin{aligned} \sup _{t \in [0, T]} \Vert v(t)\Vert _{H^{s-\sigma _1}} \le C(T, R) =: R^* \end{aligned}$$

for all solutions *v* to (3.6) with $v|_{t = 0} \in B_R$.

Suppose that $A \in {\mathcal {B}}_{H^{s-\sigma _1}}$ is a Borel set in $H^{s-\sigma _1}$ such that $\mu _s(A) = 0$. Given $R>0$, let $R^*$ be as in (5.9). Then, from (5.9), we have

5.10 $$\begin{aligned} \Psi (t) (A\cap B_R ) \subset B_{R^*} \end{aligned}$$

for all $t \in [0, T]$. Noting that $\mu _s(A\cap B_R) =0$, it follows from Proposition 5.3 and the result in [62] that

5.11 $$\begin{aligned} \mu _s(\Psi (t)(A\cap B_R)) =0 \end{aligned}$$

for any $0 \le t \le \tau$, where $\tau = \tau (R^*)$ is as in Proposition 5.3. In view of (5.10), we can iteratively apply Proposition 5.3 and the main result in [62] on time interval $[j \tau , (j+1) \tau ]$ and see that (5.11) holds for all $t \in [0, T]$. In particular, we have

$$\begin{aligned} \mu _s(\Psi (T)(A\cap B_R)) =0. \end{aligned}$$

Now, letting $R \rightarrow \infty$, it follows from the continuity from below of a measure that $\mu _s(\Psi (T)(A))=0$. Note that the choice of *T* was arbitrary. In view of the time reversibility of the Eq. (3.6), we conclude that $\mu _s$ is quasi-invariant under the flow of (3.6). Therefore, Theorem 1.2 follows from (5.8) with Lemmas 4.1, 4.4, and 4.5.

The remaining part of this section is devoted to the proof of Proposition 5.3. The claim (i) follows from the well-posedness of (3.6) in $H^{s-\sigma _1}$. In particular, the continuity of $\Psi (t)$ on $H^{s-\sigma _1}$ with the time reversibility implies (i). As before, from (A.12), we have the uniform growth bound:

5.12 $$\begin{aligned} \sup _{t \in [0, \tau ]} \Vert v(t)\Vert _{H^{s-\sigma _1}} \le C^{R^\theta \tau } R =: R_\tau \end{aligned}$$

for all solutions *v* to (3.6) with $v|_{t = 0} = u_0 \in B_R$. Then, the claim (ii) follows from Lemma 5.1 and the continuity of $\Psi (t)$ on $H^{s-\sigma _1}$.

We postpone the proof of the claim (iii) and first prove the claim (iv). For fixed $u_0 \in B_R \subset H^{s-\sigma _1}$ and $t \in {\mathbb {R}}$, define a map $F:H^s \rightarrow H^s$ by

$$\begin{aligned} F(h) = \Psi (t)(u_0 + h) - u_0= h + K(t) (u_0 + h) , \quad h \in H^s. \end{aligned}$$

Then, by computing a derivative of *F* at the origin, we have^3^ $DF|_{0} = Id _{H^s} + DK(t)|_{u_0}$. This is clearly a linear map. Moreover, the boundedness of $DF|_{0}$ on $H^s$ follows from the claim (iii). Note that *F* is invertible with the inverse $F^{-1}$ given by

$$\begin{aligned} F^{-1}(h) = \Psi (-t)(u_0 + h) - u_0 = h + K(-t) (u_0 + h). \end{aligned}$$

Hence, it follows from the chain rule that $DF|_{0} = Id _{H^s} + DK(t)|_{u_0}$ is invertible. Moreover, we have

5.13 $$\begin{aligned} (DF|_{0})^{-1} = Id _{H^s} + DK(-t)|_{u_0}. \end{aligned}$$

Hence, we proved the claim (iv) except for the boundedness of $(DF|_{0})^{-1}$. We will prove the boundedness of $(DF|_{0})^{-1}$ at the end of this section.

Next, we prove the claim (iii). In the following, we will prove that $DK(t)|_{u_0}$ is Hilbert-Schmidt on $H^s$ for $u_0 \in B_R \subset H^{s-\sigma _1}$ as long as $t = t(R) \ll 1$. Given $u_0 \in B_R \subset H^{s-\sigma _1}$, let *v* be the global solution to (3.6) with $v|_{t = 0} = u_0$.

We first introduce some notations. Given a multilinear^4^ expression ${\mathcal {M}}(v, v, \ldots , v)$, we use ${\mathcal {M}}(v^*, v^*, \ldots , v^*)$ to denote the sum of the form ${\mathcal {M}}(w, v, \ldots ) + {\mathcal {M}}(v, w,v, \ldots )$, where each multilinear term has exactly one factor of *w* and the remaining arguments are *v*. For example, we have

$$\begin{aligned} v^*_{n_1}(t)\overline{v^*_{n_2}(t)}v^*_{n_3}(t) = w_{n_1}(t)\overline{v_{n_2}(t)}v_{n_3}(t) + v_{n_1}(t)\overline{w_{n_2}(t)}v_{n_3}(t) +v_{n_1}(t)\overline{v_{n_2}(t)}w_{n_3}(t). \end{aligned}$$

We use a similar convention for multilinear expressions in *v*(0). In this case, we use ${\mathcal {M}}(v^*(0), v^*(0), \ldots )$ to denote the sum of the form ${\mathcal {M}}(w(0), v(0), \ldots ) + {\mathcal {M}}(v(0), w(0),v(0), \ldots )$, where each multilinear term has exactly one factor of *w*(0) and the remaining arguments are *v*(0).

Let *w*(*t*) be a solution to the following linear equation:

5.14 $$\begin{aligned} {\left\{ \begin{array}{ll} \displaystyle \partial _tw_n = -i \sum _{\Gamma (n)} e^{-i \phi ({\bar{n}}) t} v^*_{n_1}\overline{v^*_{n_2}}v^*_{n_3} + i |v^*_n|^2 v^*_n\\ w|_{t= 0} = w(0). \end{array}\right. } \end{aligned}$$

Given $(m_1, m_2, m_3) \in {\mathbb {Z}}^3$ and $n \in {\mathbb {Z}}$, we use the following shorthand notation:

5.15 $$\begin{aligned} ({\bar{m}}, n) := (m_1, m_2, m_3, n). \end{aligned}$$

Then, by a direct computation with (3.6), (5.1), and (5.2), we have

$$\begin{aligned}&{\mathcal {F}}\big [DK(t)|_{u_0}(w(0))\big ](n) = i \int _0^t |v^*_n(t')|^2v^*_{n}(t') dt' \nonumber \\&\quad + \sum _{\Gamma ( n)} \frac{ e^{-i \phi ( {\bar{n}}) t} }{\phi ({\bar{n}})} v^*_{n_1}(t)\overline{v^*_{n_2}(t)}v^*_{n_3}(t) - \sum _{\Gamma ( n)} \frac{ 1}{\phi ({\bar{n}})} v^*_{n_1}(0)\overline{v^*_{n_2}(0)}v^*_{n_3}(0) \nonumber \\&\quad +2 i \int _0^t \sum _{\begin{array}{c} (n_1, n_2, n_3) \in \Gamma (n)\\ (m_1, m_2, m_3) \in \Gamma (n_1) \end{array}} \frac{ e^{-i \phi ({\bar{n}})t' -i \phi ({\bar{m}}, n_1)t'} }{\phi ({\bar{n}})} v^*_{m_1}\overline{ v^*_{m_2}} v^*_{m_3}\overline{v^*_{n_2}}v^*_{n_3}(t') dt' \nonumber \\&\quad -2 i \int _0^t \sum _{\Gamma ( n)} \frac{ e^{-i \phi ({\bar{n}})t' } }{\phi ({\bar{n}})} |v^*_{n_1}|^2v^*_{n_1}\overline{v^*_{n_2}}v^*_{n_3}(t') dt' \nonumber \\&\quad - i \int _0^t \sum _{\begin{array}{c} (n_1, n_2, n_3) \in \Gamma (n)\\ (m_1, m_2, m_3) \in \Gamma (n_2) \end{array}} \frac{ e^{-i \phi ({\bar{n}}) t' +i \phi ({\bar{m}}, n_2) t'} }{\phi ({\bar{n}})} v^*_{n_1}\overline{v^*_{m_1}}v^*_{m_2}\overline{v^*_{m_3}}v^*_{n_3}(t') dt' \nonumber \\&\quad + i \int _0^t \sum _{\Gamma ( n)} \frac{ e^{-i \phi ({\bar{n}}) t' } }{\phi ({\bar{n}})} v^*_{n_1}|v^*_{n_2}|^2\overline{v^*_{n_2}}v^*_{n_3}(t') dt', \end{aligned}$$

where $\phi ({\bar{n}})$ and $\Gamma (n)$ are as in (3.7) and (3.8), respectively.

Fix $\sigma _2 > \frac{1}{2}$ (to be chosen later) and write

$$\begin{aligned} DK(t)|_{u_0}(w(0)) = \langle \partial _x \rangle ^{-\sigma _2} \circ A_t(w(0)) \end{aligned}$$

where $A_t(w(0))$ is given by

5.16 $$\begin{aligned}&{\mathcal {F}}\big [A_t(w(0))\big ](n) = i \int _0^t \langle n \rangle ^{\sigma _2} |v^*_n(t')|^2v^*_{n}(t') dt' \nonumber \\&\quad + \sum _{\Gamma ( n)} \frac{ e^{-i \phi ( {\bar{n}}) t} }{\phi ({\bar{n}})} \langle n \rangle ^{\sigma _2} v^*_{n_1}(t)\overline{v^*_{n_2}(t)}v^*_{n_3}(t) - \sum _{\Gamma (n)} \frac{ 1}{\phi ({\bar{n}})} \langle n \rangle ^{\sigma _2} v^*_{n_1}(0)\overline{v^*_{n_2}(0)}v^*_{n_3}(0) \nonumber \\&\quad +2 i \int _0^t \sum _{\begin{array}{c} (n_1, n_2, n_3) \in \Gamma (n)\\ (m_1, m_2, m_3) \in \Gamma (n_1) \end{array}} \frac{ e^{-i \phi ({\bar{n}})t' -i \phi ({\bar{m}}, n_1)t'} }{\phi ({\bar{n}})} \langle n \rangle ^{\sigma _2} v^*_{m_1}\overline{ v^*_{m_2}} v^*_{m_3}\overline{v^*_{n_2}}v^*_{n_3}(t') dt' \nonumber \\&\quad -2 i \int _0^t \sum _{\Gamma ( n)} \frac{ e^{-i \phi (\bar{n})t' } }{\phi ({\bar{n}})} \langle n \rangle ^{\sigma _2} |v^*_{n_1}|^2 v^*_{n_1}\overline{v^*_{n_2}}v^*_{n_3}(t') dt' \nonumber \\&\quad - i \int _0^t \sum _{\begin{array}{c} (n_1, n_2, n_3) \in \Gamma (n)\\ (m_1, m_2, m_3) \in \Gamma (n_2) \end{array}} \frac{ e^{-i \phi ({\bar{n}}) t' +i \phi ({\bar{m}}, n_2) t'} }{\phi ({\bar{n}})} \langle n \rangle ^{\sigma _2} v^*_{n_1}\overline{v^*_{m_1}}v^*_{m_2}\overline{v^*_{m_3}}v^*_{n_3}(t') dt' \nonumber \\&\quad + i \int _0^t \sum _{\Gamma ( n)} \frac{ e^{-i \phi ({\bar{n}}) t' } }{\phi ({\bar{n}})} \langle n \rangle ^{\sigma _2} v^*_{n_1}|v^*_{n_2}|^2\overline{v^*_{n_2}}v^*_{n_3}(t') dt', \end{aligned}$$

Note that $\langle \partial _x \rangle ^{-\sigma _2}$ is a Hilbert–Schmidt operator on $H^s$. Thus, if we prove that $A_t$ is bounded on $H^s$, then it follows that $DK(t)|_{u_0}$ is Hilbert-Schmidt on $H^s$. Hence, we focus on proving the boundedness of $A_t$ on $H^s$ in the following.

Let $$t \in [0, 1]$$. Given $$ s > 1$$, choose $$\sigma _1, \sigma _2 > \frac{1}{2}$$ such that

5.17 $$\begin{aligned} s- \sigma _1 > \tfrac{1}{2}, \quad \tfrac{s+\sigma _2}{3} \le s - \sigma _1, \quad \text {and} \quad s + \sigma _2 - 2 \le s - \sigma _1. \end{aligned}$$

Applying Young’s inequality and $\ell ^2_n \subset \ell ^6_n$ to (5.16) with Lemma 3.1 and (5.17), we have

5.18 $$\begin{aligned}&\Vert A_t(w(0))\Vert _{H^s} \lesssim \sup _{t' \in [0, t]} \bigg \{ \Vert v(t')\Vert ^2_{H^\frac{s+\sigma _2}{3}}\Vert w(t')\Vert _{H^\frac{s+\sigma _2}{3}} + \Vert v(t')\Vert ^2_{H^{s-\sigma _1}} \Vert w(t')\Vert _{H^{s-\sigma _1}}\nonumber \\&\quad + \Vert v(t')\Vert ^4_{H^{s-\sigma _1}} \Vert w(t')\Vert _{H^{s-\sigma _1}} \bigg \}. \end{aligned}$$

Given $\tau > 0$, it follows from (A.12) that

5.19 $$\begin{aligned} \sup _{t \in [0, \tau ]}\Vert v(t)\Vert _{H^{s - \sigma _1 }} \le C(R). \end{aligned}$$

Then, from (5.14) and (5.19) with (5.17), we have

$$\begin{aligned} \sup _{t \in [0, \tau ]}\Vert w(t)\Vert _{H^{s - \sigma _1 }}&\le \Vert w(0)\Vert _{H^{s}} + C \tau \sup _{t \in [0, \tau ]}\Vert v(t)\Vert ^2_{H^{s - \sigma _1 }}\Vert w(t)\Vert _{H^{s - \sigma _1 }}\nonumber \\&\le \Vert w(0)\Vert _{H^{s}} + C(R) \tau \sup _{t \in [0, \tau ]}\Vert w(t)\Vert _{H^{s - \sigma _1 }}. \end{aligned}$$

In particular, by choosing $\tau = \tau (R) > 0$ sufficiently small, we obtain

5.20 $$\begin{aligned} \sup _{t \in [0, \tau ]}\Vert w(t')\Vert _{H^{s - \sigma _1 }} \lesssim \Vert w(0)\Vert _{H^{s}}. \end{aligned}$$

Finally, it follows from (5.18), (5.19), and (5.20) with (5.17) that

$$\begin{aligned} \Vert A_t(w(0))\Vert _{H^s} \le C(R) \Vert w(0)\Vert _{H^{s}}. \end{aligned}$$

Therefore, $A_t$ is bounded on $H^s$ and hence $DK(t)|_{u_0}$ is a Hilbert–Schmidt operator on $H^s$ for all $t \in [0, \tau ]$. The second claim in (iii) basically follows from the continuous dependence of (3.6) and (5.14) (in *v*) and thus we omit details.

It remains to prove the boundedness of $(DF|_{0})^{-1} = (Id _{H^s} + DK(t)|_{u_0})^{-1}$ By the time reversibility of the equation and (5.13), the argument above shows that $(DF|_0)^{-1} - \text {Id}_{H^s}$ is Hilbert–Schmidt on $H^s$ by choosing $\tau = \tau (R)$ sufficiently small. In particular, $(DF|_0)^{-1}$ is bounded on $H^s$. This completes the proof of Proposition 5.3.

#### Remark 5.4

The condition $s > 1$ is necessary for this argument. In estimating the resonant term, i.e. the first term in (5.16) by the $H^{s-\sigma _1}$-norms of its arguments, we need to use the second condition $\tfrac{s+\sigma _2}{3} \le s - \sigma _1$ in (5.17). Thus, we must have

$$\begin{aligned} s \ge \frac{3\sigma _1 + \sigma _2}{2} > 1, \end{aligned}$$

since $$\sigma _1, \sigma _2 > \frac{1}{2}$$.

## Proof of Theorem 1.2: $s > \frac{3}{4}$

In this section, we present the proof of Theorem 1.2 for $s > \frac{3}{4}$. The basic structure of our argument follows the argument introduced in [70] by the second author in the context of the (generalized) BBM equation, with one importance difference. While the energy estimate in [70] was carried out on the $H^s$-norm of solutions (to the truncated equations), we carry out our energy estimate on a modified energy. This introduction of a modified energy is necessary to exhibit a hidden nonlinear smoothing, exploiting the dispersion of the equation. See Proposition 6.1 below. This, in turn, forces us to work with the weighted Gaussian measure $\rho _{s, N, r, t}$ and $\rho _{s, r, t}$ adapted to this modified energy, instead of the Gaussian measure $\mu _{s, r}$ with an $L^2$-cutoff. See (6.21) and (6.22) below for the definitions of $\rho _{s, N, r, t}$ and $\rho _{s, r, t}$. Lastly, we point out that this usage of the modified energy is close to the spirit of higher order modified energies in the *I*-method introduced by Colliander–Keel–Staffilani–Takaoka–Tao [22, 23].

As in Sect. 5, we carry out our analysis on (3.6). Let us first introduce the following truncated approximation to (3.6):

6.1 $$\begin{aligned} \partial _tv_n&= {\mathbf {1}}_{|n|\le N}\bigg \{-i \sum _{\Gamma _N(n)} e^{-i \phi (\bar{n}) t} v_{n_1}\overline{v_{n_2}}v_{n_3} + i |v_n|^2 v_n\bigg \}, \end{aligned}$$

where $\Gamma _N(n)$ is defined by

6.2 $$\begin{aligned} \Gamma _N(n)&= \Gamma (n) \cap \{ (n_1, n_2, n_3) \in {\mathbb {Z}}^3: |n_j|\le N\}\nonumber \\&= \{(n_1, n_2, n_3) \in {\mathbb {Z}}^3:\, n = n_1 - n_2 + n_3, \, n_1, n_3, \ne n, \text { and } |n_j| \le N\}. \end{aligned}$$

A major part of this section is devoted to the study of the dynamical properties of (6.1). Note that (6.1) is an infinite dimensional system ODEs for the Fourier coefficients $\{ v_n \}_{n \in {\mathbb {Z}}}$, where the flow is constant on the high frequencies $\{|n|> N\}$.

We also consider the following finite dimensional system of ODEs:

6.3 $$\begin{aligned} \partial _tv_n = -i \sum _{\Gamma _N(n)} e^{-i \phi ({\bar{n}}) t} v_{n_1}\overline{v_{n_2}}v_{n_3} + i |v_n|^2 v_n, \quad |n| \le N. \end{aligned}$$

Given $t, \tau \in {\mathbb {R}}$, denote by $\Psi _N(t, \tau )$ and $\widetilde{\Psi }_N(t, \tau )$ the solution maps of (6.1) and (6.3), sending initial data at time $\tau$ to solutions at time *t*, respectively. For simplicity, we set

6.4 $$\begin{aligned} \Psi _N(t) = \Psi _N(t, 0) \quad \text {and} \quad \widetilde{\Psi }_N(t) = \widetilde{\Psi }_N(t, 0) \end{aligned}$$

when $\tau = 0$. Then, we have the following relations:

6.5 $$\begin{aligned} \Psi _N(t, \tau ) = \widetilde{\Psi }_N (t, \tau ) {\mathbf {P}}_{\le N} + {\mathbf {P}}_{>N} \quad \text {and} \quad {\mathbf {P}}_{\le N} \Psi _N(t, \tau ) = \widetilde{\Psi }_N (t, \tau ) {\mathbf {P}}_{\le N}. \end{aligned}$$

### Energy estimate

In this subsection, we establish a key energy estimate. Before stating the main proposition, let us first perform a preliminary computation. Given a smooth solution *u* to (1.1), let *v* be as in (3.4). Then, from (3.6), we have

6.6 $$\begin{aligned} \frac{d}{dt} \Vert u (t) \Vert _{H^s}^2 = \frac{d}{dt} \Vert v(t) \Vert _{H^s}^2 = - 2{\hbox {Re }}i \sum _{n \in {\mathbb {Z}}} \sum _{\Gamma (n)} e^{ - i \phi ({\bar{n}}) t} \langle n \rangle ^{2s} v_{n_1} \overline{v_{n_2}} v_{n_3} \overline{v_n}. \end{aligned}$$

Then, differentiating by parts, i.e. integrating by parts without an integral sign,^5^ we obtain

6.7 $$\begin{aligned} \frac{d}{dt} \Vert v(t) \Vert _{H^s}^2&= 2{\hbox {Re }}\frac{d}{dt} \bigg [ \sum _{n \in {\mathbb {Z}}} \sum _{\Gamma (n)} \frac{e^{ - i \phi ({\bar{n}}) t}}{ \phi ({\bar{n}})} \langle n \rangle ^{2s} v_{n_1} \overline{v_{n_2}} v_{n_3} \overline{v_n}\bigg ]\nonumber \\&\quad - 2{\hbox {Re }}\sum _{n \in {\mathbb {Z}}} \sum _{\Gamma (n)} \frac{e^{ - i \phi ({\bar{n}}) t}}{ \phi ({\bar{n}})} \langle n \rangle ^{2s} \partial _t(v_{n_1} \overline{v_{n_2}} v_{n_3} \overline{v_n}). \end{aligned}$$

This motivates us to define the following quantity. Given $s > \frac{1}{2}$, define the modified energy $E_t(v)$ by

6.8 $$\begin{aligned} E_t(v)&= \Vert v\Vert _{H^s}^2 - 2{\hbox {Re }}\sum _{n \in {\mathbb {Z}}} \sum _{\Gamma (n)} \frac{e^{ - i \phi ({\bar{n}}) t}}{ \phi ({\bar{n}})} \langle n \rangle ^{2s} v_{n_1} \overline{v_{n_2}} v_{n_3} \overline{v_n} \nonumber \\&=: \Vert v\Vert _{H^s}^2 + R_t(v). \end{aligned}$$

Then, we have the following energy estimate.

#### Proposition 6.1

Let $s > \frac{3}{4}$. Then, for any sufficiently small $\varepsilon > 0$, there exist small $\theta > 0$ and $C>0$ such that

6.9 $$\begin{aligned} \bigg |\frac{d}{dt} E_t({\mathbf {P}}_{\le N} v) \bigg | \le C \Vert v \Vert _{L^2}^{4+ \theta } \Vert v\Vert _{H^{s - \frac{1}{2} - \varepsilon }}^{2-\theta }, \end{aligned}$$

for all $N \in {\mathbb {N}}$ and any solution *v* to (6.1), uniformly in $t \in {\mathbb {R}}$.

Recall that the probability measures $\mu _s$ and $\mu _{s, r}$ defined in (1.5) and (2.5) are supported on $H^{s - \frac{1}{2} -\varepsilon }({\mathbb {T}})$ for any $\varepsilon > 0$, while we have $\Vert v \Vert _{L^2} \le r$ in the support of $\mu _{s, r}$.

Before proceeding to the proof of this proposition, recall the following arithmetic fact [39]. Given $n \in {\mathbb {N}}$, the number *d*(*n*) of the divisors of *n* satisfies

6.10 $$\begin{aligned} d(n) \le C_\delta n^\delta \end{aligned}$$

for any $$\delta > 0$$.

#### Proof

Let *v* be a solution to (6.1). Then, from (6.7) and (6.8) with (6.1), we have

6.11 $$\begin{aligned} \frac{d}{dt} E_t({\mathbf {P}}_{\le N} v)&= {\mathcal {N}}_1(v)+{\mathcal {R}}_1(v) + {\mathcal {N}}_2(v)+{\mathcal {R}}_2(v) +{\mathcal {N}}_3(v)+{\mathcal {R}}_3(v), \end{aligned}$$

where ${\mathcal {N}}_j(v)$ and ${\mathcal {R}}_j(v)$, $j = 1, 2, 3$, are defined by

6.12 $$\begin{aligned} {\mathcal {N}}_1(v)(t)&:= 4{\hbox {Re }}i \sum _{|n|\le N} \sum _{\begin{array}{c} (n_1, n_2, n_3) \in \Gamma _N(n)\\ (m_1, m_2, m_3) \in \Gamma _N(n_1) \end{array}} \frac{ e^{-i \phi ({\bar{n}})t -i \phi ({\bar{m}}, n_1)t} }{\phi ({\bar{n}})} \langle n \rangle ^{2s} v_{m_1}\overline{ v_{m_2}} v_{m_3}\overline{v_{n_2}}v_{n_3} \overline{v_n} \nonumber \\ {\mathcal {R}}_1(v)(t)&:= - 4 {\hbox {Re }}i \sum _{|n|\le N} \sum _{\Gamma _N( n)} \frac{ e^{-i \phi (\bar{n})t } }{\phi ({\bar{n}})} \langle n \rangle ^{2s} |v_{n_1}|^2v_{n_1}\overline{v_{n_2}}v_{n_3}\overline{v_n} \nonumber \\ {\mathcal {N}}_2(v)(t)&:= - 2{\hbox {Re }}i \sum _{|n|\le N} \sum _{\begin{array}{c} (n_1, n_2, n_3) \in \Gamma _N(n)\\ (m_1, m_2, m_3) \in \Gamma _N(n_2) \end{array}} \frac{ e^{-i \phi ({\bar{n}}) t +i \phi ({\bar{m}}, n_2) t} }{\phi ({\bar{n}})} \langle n \rangle ^{2s} v_{n_1}\overline{v_{m_1}}v_{m_2}\overline{v_{m_3}}v_{n_3}\overline{v_n} \nonumber \\ {\mathcal {R}}_2(v)(t)&:= 2{\hbox {Re }}i \sum _{|n|\le N} \sum _{\Gamma _N( n)} \frac{ e^{-i \phi ({\bar{n}}) t } }{\phi ({\bar{n}})} \langle n \rangle ^{2s} v_{n_1}|v_{n_2}|^2\overline{v_{n_2}}v_{n_3}\overline{v_n} \nonumber \\ {\mathcal {N}}_3(v)(t)&:= - 2{\hbox {Re }}i \sum _{|n|\le N} \sum _{\begin{array}{c} (n_1, n_2, n_3) \in \Gamma _N( n)\\ (m_1, m_2, m_3) \in \Gamma _N( n) \end{array}} \frac{ e^{-i \phi ({\bar{n}}) t +i \phi ({\bar{m}}, n) t} }{\phi ({\bar{n}})} \langle n \rangle ^{2s} v_{n_1} \overline{v_{n_2}}v_{n_3} \overline{v_{m_1}}v_{m_2}\overline{v_{m_3}} \nonumber \\ {\mathcal {R}}_3(v)(t)&:= 2{\hbox {Re }}i \sum _{|n|\le N} \sum _{\Gamma _N( n)} \frac{ e^{-i \phi ({\bar{n}}) t } }{\phi ({\bar{n}})} \langle n \rangle ^{2s} v_{n_1}\overline{v_{n_2}}v_{n_3}|v_{n}|^2\overline{v_n}. \end{aligned}$$

Here, $({\bar{m}}, n) = (m_1, m_2, m_3, n)$ and $({\bar{m}}, n_j) = (m_1, m_2, m_3, n_j)$ are as in (5.15). For simplicity of the presentation, we drop the restriction on the summations in (6.12) with the understanding that $v_n = 0$ for $|n|> N$. Moreover, we can assume that all the Fourier coefficients are non-negative. In the following, we establish uniform (in *t*) estimates for these multilinear terms ${\mathcal {N}}_j$ and ${\mathcal {R}}_j$, $j = 1, 2, 3$. For simplicity, we suppress the *t*-dependence with the understanding that all the estimates hold with implicit constants independent of $t \in {\mathbb {R}}$.

Given $n, \mu \in {\mathbb {Z}}$, define $\Gamma (n, \mu )$ by

$$\begin{aligned} \Gamma (n, \mu )&:=\Gamma (n) \cap \{ (n_1, n_2, n_3) \in {\mathbb {Z}}^3: \mu = (n-n_1)(n-n_3)\} \nonumber \\&= \{ (n_1, n_2, n_3) \in {\mathbb {Z}}^3: n = n_1 - n_2 + n_3, \ \nonumber \\&\quad n_1, n_3 \ne n,\ \mu = (n-n_1)(n-n_3)\}. \end{aligned}$$

Then, given $\delta > 0$, it follows from the divisor counting estimate (6.10) that

6.13 $$\begin{aligned} \# \Gamma (n, \mu )= \sum _{\Gamma (n, \mu )} 1 \le C_\delta |\mu |^\delta . \end{aligned}$$

In the following, we use (6.13) to estimate ${\mathcal {N}}_j(v)$ and ${\mathcal {R}}_j(v)$, $j = 1, 2, 3$. For simplicity of the presentation, we drop multiplicative constants depending on $\delta > 0$.

We now estimate $${\mathcal {N}}_1(v)$$. We first consider the case $$s <1 $$. By Sobolev’s inequality and interpolation, we have

6.14 $$\begin{aligned} \bigg \Vert \sum _{\Gamma (n_1)} v_{m_1}\overline{ v_{m_2}} v_{m_3}\bigg \Vert _{\ell ^\infty _{n_1}}&\le \Vert {\mathcal {F}}^{-1}(|\widehat{v}_n|)\Vert _{L^3}^3 \lesssim \Vert v\Vert _{H^\frac{1}{6}}^3 \lesssim \Vert v \Vert _{L^2}^{1+\theta } \Vert v\Vert _{H^{\frac{1}{4}+\gamma }}^{2-\theta } \end{aligned}$$

for small $$\gamma > 0$$ and some $$\theta = \theta (\gamma ) >0$$. Then, by Lemma 3.1 and Cauchy–Schwarz inequality (in *n* and then in $$ n_2, n_3$$) with (6.14), we have

6.15 $$\begin{aligned} |{\mathcal {N}}_1(v)|&\lesssim \sum _{n \in {\mathbb {Z}}} \sum _{\mu \ne 0} \sum _{\Gamma (n, \mu )} \frac{1}{|\mu |n_{\max }^{2-2s}} v_{n_2}v_{n_3} v_n \bigg \Vert \sum _{\Gamma (n_1)} v_{m_1}\overline{ v_{m_2}} v_{m_3}\bigg \Vert _{\ell ^\infty _{n_1}} \nonumber \\&\le \Vert v \Vert _{L^2}^{2+\theta } \Vert v\Vert _{H^{\frac{1}{4}+\gamma }}^{2-\theta }\nonumber \\&\quad \times \Bigg \{ \sum _{n \in {\mathbb {Z}}} \bigg (\sum _{\mu \ne 0} \frac{1}{|\mu |^{1+2\delta }} \sum _{\Gamma ( n, \mu )} 1\bigg ) \bigg (\sum _{\Gamma (n)} \frac{v_{n_2}^2 v_{n_3} ^2 }{|(n_2 - n_3)(n-n_3)|^{1-2\delta }n_{\max }^{4-4s}}\bigg )\Bigg \}^{\frac{1}{2}} \end{aligned}$$

for small $\gamma , \delta > 0$ such that $5 - 4s - 2\delta > 1$, where $n_{\max } : = \max (|n|, |n_1|, |n_2|, |n_3|)$. From the divisor counting argument (6.13), we have

6.16 $$\begin{aligned} |{\mathcal {N}}_1(v)|&\lesssim \Vert v \Vert _{L^2}^{2+\theta } \Vert v\Vert _{H^{\frac{1}{4}+\gamma }}^{2-\theta } \Bigg \{ \sum _{n \in {\mathbb {Z}}} \bigg (\sum _{\mu \ne 0} \frac{1}{\mu ^{1+2\delta }} |\mu |^{\delta }\bigg ) \bigg (\sum _{\Gamma (n)} \frac{ v_{n_2}^2 v_{n_3} ^2 }{|(n - n_1)(n-n_3)|^{1-2\delta }n_{\max }^{4-4s}}\bigg )\Bigg \}^{\frac{1}{2}} \nonumber \\&\lesssim \Vert v \Vert _{L^2}^{2+\theta } \Vert v\Vert _{H^{\frac{1}{4}+\gamma }}^{2-\theta } \Bigg \{ \sum _{n_2, n_3 \in {\mathbb {Z}}} v_{n_2}^2 v_{n_3} ^2 \sum _{n \ne n_3} \frac{1}{|n-n_3|^{1-2\delta }\langle n \rangle ^{4-4s}}\Bigg \}^{\frac{1}{2}} \nonumber \\&\lesssim \Vert v \Vert _{L^2}^{4+\theta } \Vert v\Vert _{H^{s - \frac{1}{2} - \varepsilon }}^{2-\theta } \end{aligned}$$

for sufficiently small $\delta , \varepsilon , \gamma > 0$, provided that $s > \frac{3}{4}$.

Next, we consider the case $s \ge 1$. Note that for $(n_1, n_2, n_3) \in \Gamma (n)$, we have $\max _{j = 1,2, 3}|n_j|\gtrsim |n|$. First, suppose that $|n_1| \gtrsim |n|$. In this case, we use the fact that $\max _{j = 1,2, 3}|m_j|\gtrsim |n_1|$ for $(m_1, m_2, m_3) \in \Gamma (n_1)$. Without loss of generality, assume that $|m_1|\gtrsim |n_1| (\gtrsim |n|)$. Proceeding as in (6.15) and (6.16) with (6.14), Cauchy-Schwarz inequality, (6.13), and interpolation, we have

6.17 $$\begin{aligned} |{\mathcal {N}}_1(v)|&\lesssim \sum _{n \in {\mathbb {Z}}} \sum _{\mu \ne 0} \sum _{\Gamma (n, \mu )} \frac{1}{|\mu | \langle n \rangle ^{2\delta }} {v_{n_2}}v_{n_3} \langle n \rangle ^{s - 1+\delta } {v_n} \bigg \Vert \sum _{\Gamma (n_1)} \langle m_1 \rangle ^{s - 1+\delta } v_{m_1}\overline{ v_{m_2}} v_{m_3}\bigg \Vert _{\ell ^\infty _{n_1}} \nonumber \\&\lesssim \Vert v\Vert _{H^{s-1+\delta }} \Vert v\Vert _{H^{s-\frac{5}{6} +\delta }} \Vert v\Vert _{H^{\frac{1}{6}}}^2 \nonumber \\&\quad \times \Bigg \{ \sum _{n \in {\mathbb {Z}}} \bigg (\sum _{\mu \ne 0} \frac{1}{|\mu |^{1+2\delta }} \sum _{\Gamma ( n, \mu )}1 \bigg ) \bigg (\sum _{\Gamma (n)} \frac{v_{n_2}^2 v_{n_3} ^2 }{|(n - n_1)(n-n_3)|^{1-2\delta }\langle n \rangle ^{4\delta }}\bigg )\Bigg \}^{\frac{1}{2}}\nonumber \\&\lesssim \Vert v\Vert _{H^{s-1+ \delta }} \Vert v\Vert _{H^{s-\frac{5}{6} +\delta }} \Vert v\Vert _{H^{\frac{1}{6}}}^2 \nonumber \\&\quad \times \Bigg \{ \sum _{n \in {\mathbb {Z}}} \bigg (\sum _{\mu \ne 0} \frac{1}{|\mu |^{1+2\delta }} |\mu |^\delta \bigg ) \bigg (\sum _{\Gamma (n)} \frac{v_{n_2}^2 v_{n_3} ^2 }{|(n - n_1)(n-n_3)|^{1-2\delta }\langle n \rangle ^{4\delta }}\bigg )\Bigg \}^{\frac{1}{2}}\nonumber \\&\lesssim \Vert v\Vert _{H^{s-1+ \delta }} \Vert v\Vert _{H^{s-\frac{5}{6} +\delta }} \Vert v\Vert _{H^{\frac{1}{6}}}^2 \Bigg \{ \sum _{n_2, n_3 \in {\mathbb {Z}}} v_{n_2}^2 v_{n_3} ^2 \sum _{n \ne n_3} \frac{1 }{|n-n_3|^{1-2\delta }\langle n \rangle ^{4\delta }}\Bigg \}^{\frac{1}{2}}\nonumber \\&\lesssim \Vert v\Vert _{L^2}^2 \Vert v\Vert _{H^{s-1 + \delta }} \Vert v\Vert _{H^{s-\frac{5}{6} +\delta }} \Vert v\Vert _{H^{\frac{1}{6}}}^2 \lesssim \Vert v \Vert _{L^2}^{4+\theta } \Vert v\Vert _{H^{s - \frac{1}{2} - \varepsilon }}^{2-\theta } \end{aligned}$$

for sufficiently small $\delta , \varepsilon >0$ and some $\theta = \theta (s,\delta , \varepsilon ) > 0$.

Suppose that $|n_1|\ll |n|$. In this case, we have $\max (|n_2|, |n_3|) \gtrsim |n|$. Without loss of generality, assume that $|n_2 |\gtrsim |n|$. Proceeding as in (6.15) and (6.16) with (6.13) and (6.14), we have

6.18 $$\begin{aligned} |{\mathcal {N}}_1(v)|&\lesssim \sum _{n \in {\mathbb {Z}}} \sum _{\mu \ne 0} \sum _{\Gamma (n, \mu )} \frac{1}{|\mu |\langle n \rangle ^{2\delta }} \langle n_2 \rangle ^{s-1+\delta }{v_{n_2}}v_{n_3} \langle n \rangle ^{s - 1+\delta } {v_n} \bigg \Vert \sum _{\Gamma (n_1)} v_{m_1}\overline{ v_{m_2}} v_{m_3}\bigg \Vert _{\ell ^\infty _{n_1}} \nonumber \\&\lesssim \Vert v\Vert _{H^{s-1+\delta }}\Vert v\Vert _{H^\frac{1}{6}}^3 \Bigg \{ \sum _{n \in {\mathbb {Z}}} \bigg (\sum _{\mu \ne 0} \frac{1}{|\mu |^{1+2\delta }} |\mu |^\delta \bigg ) \bigg (\sum _{\Gamma (n)} \frac{\langle n_2 \rangle ^{2(s-1+\delta )}v_{n_2}^2 v_{n_3} ^2 }{|(n - n_1)(n-n_3)|^{1-2\delta }\langle n \rangle ^{4\delta }}\bigg )\Bigg \}^{\frac{1}{2}}\nonumber \\&\lesssim \Vert v\Vert _{H^{s-1+\delta }}\Vert v\Vert _{H^\frac{1}{6}}^3 \Bigg \{ \sum _{n_2, n_3 \in {\mathbb {Z}}} \langle n_2 \rangle ^{2(s-1+\delta )} v_{n_2}^2 v_{n_3} ^2 \sum _{n \ne n_3} \frac{1 }{|n-n_3|^{1-2\delta }\langle n \rangle ^{4\delta }}\Bigg \}^{\frac{1}{2}}\nonumber \\&\lesssim \Vert v\Vert _{L^2} \Vert v\Vert _{H^{s-1+\delta }}^2 \Vert v\Vert _{H^{\frac{1}{6}}}^3 \lesssim \Vert v\Vert _{L^2}^{4+\theta } \Vert v\Vert _{H^{s-\frac{1}{2} -\varepsilon }}^{2-\theta } \end{aligned}$$

for sufficiently small $\delta , \varepsilon >0$ and some $\theta = \theta (s, \delta , \varepsilon ) > 0$.

Noting that $\mu = (n-n_1) (n - n_3) = (n_2 - n_1) (n_2 - n_3)$ under $n = n_1 - n_2 + n_3$, we can estimate ${\mathcal {N}}_2(v)$ and ${\mathcal {N}}_3(v)$ in a similar manner.

Next, we estimate ${\mathcal {R}}_1(v)$. The remaining terms ${\mathcal {R}}_2(v)$ and ${\mathcal {R}}_3(v)$ can be estimated in a similar manner. Without loss of generality, suppose that $|n_3|\gtrsim |n|$. From Lemma 3.1 and the divisor counting argument (6.13), we have

6.19 $$\begin{aligned} |{\mathcal {R}}_1(v)|&\lesssim \Vert v\Vert _{H^{s-1+\delta }} \Bigg \{ \sum _{n \in {\mathbb {Z}}} \bigg (\sum _{\mu \ne 0} \frac{1}{\mu ^{1+2\delta }} |\mu |^{\delta }\bigg ) \bigg (\sum _{\Gamma (n)} \frac{ v_{n_1}^6 v_{n_2}^2 \langle n_3 \rangle ^{2{(s-1+\delta )}} v_{n_3} ^2 }{|(n - n_1)(n-n_3)|^{1-2\delta }\langle n \rangle ^{4\delta }}\bigg )\Bigg \}^{\frac{1}{2}} \nonumber \\&\lesssim \Vert v \Vert _{L^2}^{4} \Vert v\Vert _{H^{s - 1+\delta }}^{2} \lesssim \Vert v \Vert _{L^2}^{4+ \theta } \Vert v\Vert _{H^{s - \frac{1}{2} - \varepsilon }}^{2-\theta } \end{aligned}$$

for sufficiently small $\delta , \varepsilon >0$ and some $\theta = \theta (s, \delta , \varepsilon ) > 0$.

Therefore, (6.9) follows from (6.16), (6.17), (6.18), and (6.19). This completes the proof of Proposition 6.1. $\square$

### Weighted Gaussian measures

Our main goal in this subsection is to define weighted Gaussian measures adapted to the modified energy $E_t({\mathbf {P}}_{\le N} v)$ and $E_t(v)$ defined in the previous section. Given $N \in {\mathbb {N}}$ and $r>0$, define $F_{N, r, t}(v)$ and $F_{r, t}(v)$ by

6.20 $$\begin{aligned} F_{N, r, t}(v) = {\mathbf {1}}_{\{ \Vert v\Vert _{L^2 } \le r\}} e^{-\frac{1}{2} R_t({\mathbf {P}}_{\le N} v)} \quad \text {and}\quad F_{r, t}(v) = {\mathbf {1}}_{\{ \Vert v\Vert _{L^2 } \le r\}} e^{-\frac{1}{2} R_t( v)}, \end{aligned}$$

where $R_t$ is defined in (6.8). Then, we would like to construct probability measures $\rho _{s, N, r, t}$ and $\rho _{s, r, t}$ of the form:^6^

6.21 $$\begin{aligned} d \rho _{s, N, r, t}&= \text {``}Z_{s, N, r}^{-1} {\mathbf {1}}_{\{ \Vert v\Vert _{L^2 } \le r\}} e^{- \frac{1}{2} E_t({\mathbf {P}}_{\le N} v)} dv\text {''} \nonumber \\&= Z_{s, N, r}^{-1} F_{N, r, t} d\mu _s \end{aligned}$$

and

6.22 $$\begin{aligned} d \rho _{s, r, t}&= \text {``}Z_{s, r}^{-1} {\mathbf {1}}_{\{ \Vert v\Vert _{L^2 } \le r\}} e^{- \frac{1}{2} E_t( v)} dv\text {''} \nonumber \\&= Z_{s, r}^{-1} F_{r, t} d\mu _s. \end{aligned}$$

The following proposition shows that they are indeed well defined probability measures on $H^{s-\frac{1}{2} -\varepsilon }({\mathbb {T}})$, $\varepsilon > 0$.

#### Proposition 6.2

Let $s > \frac{1}{2}$ and $r > 0$. Then, $F_{N, r, t}(v) \in L^p(\mu _s)$ for any $p \ge 1$ with a uniform bound in $N \in {\mathbb {N}}$ and $t \in {\mathbb {R}}$, depending only on $p\ge 1$ and $r > 0$. Moreover, for any finite $p \ge 1$, $F_{N, r, t}(v)$ converges to $F_{r, t}(v)$ in $L^p (\mu _s)$, uniformly in $t\in {\mathbb {R}}$, as $N \rightarrow \infty$.

In the following, we restrict our attention to $s > \frac{1}{2}$. Hence, we view $\rho _{s, N, r, t}$ and $\rho _{s, r, t}$ as probability measures on $L^2({\mathbb {T}})$.

Let $\mu _{s, r}$ be as in (2.5). Then, it follows from Proposition 6.2 that $\rho _{s, r, t}$ and $\mu _{s, r}$ are mutually absolutely continuous. Moreover, we have the following ‘uniform convergence’ property of $\rho _{s, N, r, t}$ to $\rho _{s, r, t}$.

#### Corollary 6.3

Given $s > \frac{1}{2}$ and $r>0$, let $\rho _{s, N, r, t}$ and $\rho _{s, r, t}$ be as in (6.21) and (6.22). Then, for any $\gamma > 0$, there exists $N_0 \in {\mathbb {N}}$ such that

$$\begin{aligned} | \rho _{s, N, r, t}(A) - \rho _{s, r, t}(A)| < \gamma \end{aligned}$$

for any $N \ge N_0$ and any measurable set $A \subset L^2({\mathbb {T}})$, uniformly in $t\in {\mathbb {R}}$.

The proof of Proposition 6.2 follows closely Bourgain’s argument in constructing Gibbs measures [8]. We first recall the following basic tail estimate. See [58, Lemma 4.2] for a short proof.

#### Lemma 6.4

Let $\{ g_n\}_{n \in {\mathbb {N}}}$ be independent standard complex-valued Gaussian random variables. Then, there exist constant $c, C>0$ such that, for any $M\ge 1$, we have the following tail estimate:

$$\begin{aligned} P\bigg [ \Big ( \sum _{n=1}^M |g_n|^2\Big )^\frac{1}{2} \ge K \bigg ] \le e^{-cK^2}, \quad K \ge C M^\frac{1}{2} . \end{aligned}$$

#### Proof of Proposition 6.2

Fix $r> 0$. We first prove

6.23 $$\begin{aligned} \Vert F_{N, r, t} \Vert _{L^p(\mu _s)}, \, \Vert F_{r, t}\Vert _{L^p(\mu _s)} \le C_{p, r} < \infty \end{aligned}$$

for all $N \in {\mathbb {N}}$ and $t \in {\mathbb {R}}$. From the distributional characterization of the $L^p$-norm and (6.20), we have

$$\begin{aligned} \Vert F_{r, t} \Vert _{L^p(\mu _s)}^p&= p \int _0^\infty \lambda ^{p-1} \mu _s(|F_{ r, t}| > \lambda ) d\lambda \\&\le C+ p \int _e^\infty \lambda ^{p-1} \mu _s \big (|R_t(v)| \ge \log \lambda , \, \Vert v\Vert _{L^2} \le r\big ) d\lambda . \end{aligned}$$

In the following, we estimate $$\mu _s \big (|R_t(v)| \ge K, \, \Vert v\Vert _{L^2} \le r\big ) $$ for $$K \ge 1$$, using the dyadic pigeon hole principle and Lemma 6.4. Let us divide the argument into two cases: $$ s> 1$$ and $$\frac{1}{2} < s \le 1$$. Note that, while $$R_t$$ depends on $$t \in {\mathbb {R}}$$, all the estimates below hold uniformly in $$t\in {\mathbb {R}}$$.

First, suppose that $s > 1$. Then, from Lemma 3.1 and the divisor counting argument as in the proof of Proposition 6.1 (see (6.19)), we have

6.24 $$\begin{aligned} |R_t( v)| \le C_0 \Vert v\Vert _{L^2}^2 \Vert v\Vert _{H^{s-1}}^2 \le C_0 r^2 \Vert v\Vert _{H^{s-1}}^2. \end{aligned}$$

under $\Vert v\Vert _{L^2 } \le r$. Similarly, we have

6.25 $$\begin{aligned} |R_t({\mathbf {P}}_{\le M_0} v)| \le C_0 M_0^{2(s-1)} \Vert {\mathbf {P}}_{\le M_0} v\Vert _{L^2}^4 \le C_0 M_0^{2(s-1)}r^4. \end{aligned}$$

Given $K \ge 1$, choose $M_0 > 0$ such that

6.26 $$\begin{aligned} \tfrac{1}{2} K = C_0 M_0^{2(s-1)}r^4. \end{aligned}$$

For $j \in {\mathbb {N}}$, let $M_j = 2^j M_0$ and $\sigma _j = C_\varepsilon 2^{-\varepsilon j}$ for some small $\varepsilon > 0$ such that $\sum _{j \in {\mathbb {N}}} \sigma _j = \frac{1}{2}$. Then, from (6.24) and (6.25), we have

$$\begin{aligned} \mu _s \big (|R_t(v)| \ge K, \, \Vert v\Vert _{L^2} \le r\big )&\le \mu _s \big (\Vert v\Vert _{H^{s-1}}^2 \ge C_0^{-1}r^{-2} K\big ) \nonumber \\&\le \sum _{j = 1}^\infty \mu _s \big (\Vert {\mathbf {P}}_{M_j} v\Vert _{H^{s-1}}^2 \ge \sigma _j C_0^{-1}r^{-2} K\big ) \nonumber \\&\lesssim \sum _{j = 1}^\infty P\bigg [ \Big ( \sum _{|n|\sim M_j }|g_n|^2\Big )^\frac{1}{2} \gtrsim L_j \bigg ], \end{aligned}$$

where $L_j := (\sigma _j r^{-2} K)^\frac{1}{2} M_j \gtrsim M_0^{\frac{1}{2} \varepsilon } M_j^{1-\frac{1}{2} \varepsilon } \gg M_j^{\frac{1}{2}}$. Here, we used that $r^{-2} K \sim M_0^{s-1} r^2 \gtrsim 1$ in view of (6.26). Then, applying Lemma 6.4 with (6.26), we obtain

$$\begin{aligned} \mu _s \big (|R_t(v)| \ge K, \, \Vert v\Vert _{L^2} \le r\big )&\lesssim \sum _{j = 1}^\infty e^{-c L_j^2} = \sum _{j = 1}^\infty e^{-c_r 2^{(2 - \varepsilon )j} M_0^{2} K } \nonumber \\&= \sum _{j = 1}^\infty e^{-c'_r 2^{(2 - \varepsilon )j} K^{1+\frac{1}{s-1} }} \lesssim e^{-c''_r K^{1+\frac{1}{s-1} }}. \end{aligned}$$

This proves (6.23) for $F_{r, t}$ when $s > 1$. A similar argument holds for $F_{N, r, t}$ with a uniform bound in $N \in {\mathbb {N}}$.

Next, suppose that $\frac{1}{2}< s \le 1$. Proceeding with Lemma 3.1 as before, we have

6.27 $$\begin{aligned} |R_t( v)| \lesssim \Vert v\Vert _{H^{\frac{s}{2} -\frac{1}{2}}}^4 \le r^4 \end{aligned}$$

under $\Vert v\Vert _{L^2 } \le r$. Hence, (6.23) trivially follows in this case.

It remains to show that $F_{N, r, t}$ converges to $F_{r, t}$ in $L^p(\mu _s)$. It follows from a small modification of (6.24) and (6.27) that $R_t({\mathbf {P}}_{\le N} v)$ converges to $R_t( v)$ almost surely with respect to $\mu _s$, uniformly in $t\in {\mathbb {R}}$. Indeed, when $s > 1$, we have

$$\begin{aligned} |R_t( v) - R_t({\mathbf {P}}_{\le N} v)| \lesssim \Vert {\mathbf {P}}_{> N} v\Vert _{L^2} \Vert v\Vert _{L^2} \Vert v\Vert _{H^{s-1}}^2 + \Vert v\Vert _{L^2}^2 \Vert {\mathbf {P}}_{> N} v\Vert _{H^{s-1}} \Vert v\Vert _{H^{s-1}} \longrightarrow 0, \end{aligned}$$

while we have

$$\begin{aligned} |R_t( v) - R_t({\mathbf {P}}_{\le N} v)| \lesssim \Vert {\mathbf {P}}_{> N} v\Vert _{H^{\frac{s}{2} -\frac{1}{2}}} \Vert v\Vert _{H^{\frac{s}{2} -\frac{1}{2}}}^3 \longrightarrow 0, \end{aligned}$$

when $\frac{1}{2} < s\le 1$. Hence, $F_{N, r, t}$ converges to $F_{r, t}$ almost surely with respect to $\mu _s$. As a consequence of Egoroff’s theorem, we see that $F_{N, r, t}$ converges to $F_{r, t}$ almost uniformly and hence in measure (uniformly in $t \in {\mathbb {R}}$). Namely, given $\varepsilon > 0$, if we let

$$\begin{aligned} A_{N, \varepsilon , t} = \{ v \in L^2({\mathbb {T}}):\, |F_{N, r, t}(v) - F_{r, t}(v)|\le \tfrac{1}{2} \varepsilon \}, \end{aligned}$$

we have $\mu _s(A_{N, \varepsilon , t}^c) \rightarrow 0$, uniformly in $t \in {\mathbb {R}}$, as $N \rightarrow \infty$. Then, by Cauchy-Schwarz inequality and (6.23), we have

$$\begin{aligned} \Vert F_{N, r, t} - F_{r, t}\Vert _{L^p(\mu _s)}&\le \Vert (F_{N, r, t} - F_{r, t}){\mathbf {1}}_{A_{N, \varepsilon , t}}\Vert _{L^p(\mu _s)} +\Vert (F_{N, r, t} - F_{r, t}){\mathbf {1}}_{A_{N, \varepsilon , t}^c}\Vert _{L^p(\mu _s)} \\&\le \tfrac{1}{2} \varepsilon +\Vert F_{N, r, t} - F_{r, t}\Vert _{L^{2p}(\mu _s)} \Vert {\mathbf {1}}_{A_{N, \varepsilon , t}^c}\Vert _{L^{2p}(\mu _s)} \\&\le \tfrac{1}{2}\varepsilon + 2C_{2p, r} \mu _s(A_{N, \varepsilon , t}^c)^\frac{1}{2p} \\&\le \varepsilon \end{aligned}$$

for all sufficiently large $N \in {\mathbb {N}}$, uniformly in $t \in {\mathbb {R}}$. Therefore, $F_{N, r, t}$ converges to $F_{r, t}$ in $L^p(\mu _s)$ for any $p \ge 1$. $\square$

We conclude this subsection by stating a large deviation estimate on the quantity appearing in the energy estimate (Proposition 6.1).

#### Lemma 6.5

Let $\varepsilon > 0$ and $r>0$. Then, there exists $C = C(\varepsilon , r) > 0$ such that

$$\begin{aligned} \big \Vert \Vert v \Vert _{H^{s-\frac{1}{2} - \varepsilon }} \big \Vert _{L^p(\rho _{s, N, r, t})}\le C p^\frac{1}{2} \end{aligned}$$

for any $p \ge 2$, any $t \in {\mathbb {R}}$, and all sufficiently large $N \in {\mathbb {N}}$.

#### Proof

By Proposition 6.2, we have

$$\begin{aligned} \big \Vert \Vert v \Vert _{H^{s-\frac{1}{2} - \varepsilon }} \big \Vert _{L^p(\rho _{s, N, r, t})}&\le \Vert F_{N, r}\Vert _{L^{2p}(\mu _s)} \big \Vert \Vert v \Vert _{H^{s-\frac{1}{2} - \varepsilon }} \big \Vert _{L^{2p}(\mu _s)}\nonumber \\&\lesssim \bigg \Vert \Big \Vert \sum _{n \in {\mathbb {Z}}} \frac{g_n}{\langle n \rangle ^{\frac{1}{2} + \varepsilon }}e^{inx} \Big \Vert _{L^2_x} \bigg \Vert _{L^{2p}(\Omega )}\nonumber \\&\lesssim p^\frac{1}{2} \bigg \Vert \Big \Vert \sum _{n \in {\mathbb {Z}}} \frac{g_n}{\langle n \rangle ^{\frac{1}{2} + \varepsilon }}e^{inx} \Big \Vert _{L^{2}(\Omega )}\bigg \Vert _{L^2_x} \lesssim p^\frac{1}{2}. \end{aligned}$$

Here, the second to the last inequality follows from the hypercontractvity estimate due to Nelson [54, Theorem 2]. See also [17, Lemma 3.1]. $\square$

### A change-of-variable formula

In this subsection, we establish an important change-of-variable formula (Proposition 6.6). It is strongly motivated by the work [71, 72]. We closely follow the argument presented in [70].

Given $N \in {\mathbb {N}}$, let $d L_N = \prod _{|n|\le N} d\widehat{u}_n$ denote the Lebesgue measure on ${\mathbb {C}}^{2N+1}$. Then, from (6.20) and (6.21) with (2.4), we have

$$\begin{aligned} d \rho _{s, N, r, t}&= Z_{s, N, r}^{-1} {\mathbf {1}}_{\{ \Vert v\Vert _{L^2 } \le r\}} e^{-\frac{1}{2} R_t({\mathbf {P}}_{\le N} v)} d\mu _s\nonumber \\&=\widehat{Z}_{s, N, r}^{-1} {\mathbf {1}}_{\{ \Vert v\Vert _{L^2 } \le r\}} e^{-\frac{1}{2} E_t({\mathbf {P}}_{\le N} v)} dL_N \otimes d\mu _{s, N}^\perp , \end{aligned}$$

where $\widehat{Z}_{s, N, r}$ is a normalizing constant defined by^7^

$$\begin{aligned} \widehat{Z}_{s, N, r} = \int _{L^2} {\mathbf {1}}_{\{ \Vert v\Vert _{L^2 } \le r\}} e^{-\frac{1}{2} E_t( {\mathbf {P}}_{\le N} v)} d L_N \otimes d\mu _{s, N}^\perp . \end{aligned}$$

Then, we have the following change-of-variable formula:

#### Proposition 6.6

Let $s > \frac{1}{2}$, $N \in {\mathbb {N}}$, and $r > 0$. Then, we have

6.28 $$\begin{aligned} \rho _{s, N, r, t}(\Psi _N(t, \tau )(A))&= Z_{s, N, r}^{-1}\int _{\Psi _N(t, \tau )(A)} {\mathbf {1}}_{\{ \Vert v\Vert _{L^2 } \le r\}} e^{-\frac{1}{2} R_t( {\mathbf {P}}_{\le N} v)} d\mu _s(v) \nonumber \\&= \widehat{Z}_{s, N, r}^{-1}\int _{A} {\mathbf {1}}_{\{ \Vert v\Vert _{L^2 } \le r\}} e^{-\frac{1}{2} E_t( {\mathbf {P}}_{\le N} \Psi _N(t, \tau ) (v))} d L_N \otimes d\mu _{s, N}^\perp \end{aligned}$$

for any $t, \tau \in {\mathbb {R}}$ and any measurable set $A \subset L^2$.

We first state the basic invariance property of $L_N$.

#### Lemma 6.7

Let $N \in {\mathbb {N}}$. Then, the Lebesgue measure $d L_N = \prod _{|n|\le N} d\widehat{u}_n$ is invariant under the flow $\widetilde{\Psi }_N(t, \tau )$.

#### Proof

The finite dimensional system (6.3) basically corresponds to the finite dimensional Hamiltonian approximation to (1.1) under two transformations (3.2) and (3.4). Therefore, morally speaking, the lemma should follow from the inherited Hamiltonian structure and Liouville’s theorem. In the following, however, we provide a direct proof.

Write (6.3) as $\partial _tv_n = X_n$, $|n| \le N$. Then, by Liouville’s theorem, it suffices to show

$$\begin{aligned} \sum _{|n| \le N} \bigg [ \frac{\partial {\hbox {Re }}X_n}{\partial {\hbox {Re }}v_n} + \frac{\partial {\hbox {Im }}X_n}{\partial {\hbox {Im }}v_n}\bigg ] = 0, \end{aligned}$$

or equivalently,

6.29 $$\begin{aligned} \sum _{|n| \le N} \bigg [ \frac{\partial X_n}{\partial v_n} + \frac{\partial \overline{X}_n}{\partial \overline{v}_n}\bigg ] = 0. \end{aligned}$$

Note that the first sum in (6.3) does not have any contribution to (6.29) due to the frequency restriction $n_1, n_3 \ne n$. Hence, we have

$$\begin{aligned} \frac{\partial X_n}{\partial v_n} + \frac{\partial \overline{X}_n}{\partial \overline{v}_n} = 2 i |v_n|^2 -2 i |v_n|^2 = 0 \end{aligned}$$

for each $|n|\le N$. Therefore, (6.29) holds. $\square$

We now present the proof of Proposition 6.6.

#### Proof of Proposition 6.6

The first equality in (6.28) is nothing but the definition of $\rho _{s, N, r, t}$. From (6.20) and (6.21) with (6.8), we have

$$\begin{aligned} \rho _{s, N, r, t}&(\Psi _N(t, \tau )(A)) = \widehat{Z}_{s, N, r}^{-1} \int _{E_N} \int _{E_N^\perp }{\mathbf {1}}_{\Psi _N(t, \tau )(A)} (v){\mathbf {1}}_{\{ \Vert v\Vert _{L^2 } \le r\}} e^{-\frac{1}{2} E_t( {\mathbf {P}}_{\le N} v)} dL_N \otimes d\mu _{s, N}^\perp \end{aligned}$$

By Fubini’s theorem, Lemma 6.7, and (6.5) we have

$$\begin{aligned}&= \widehat{Z}_{s, N, r}^{-1} \int _{E_N^\perp } \bigg \{ \int _{E_N} {\mathbf {1}}_{\Psi _N(t, \tau )(A)} ( \widetilde{\Psi }_N(t, \tau )( {\mathbf {P}}_{\le N} v) + {\mathbf {P}}_{>N} v) \nonumber \\&\quad \times {\mathbf {1}}_{\{ \Vert \widetilde{\Psi }_N(t, \tau ) ({\mathbf {P}}_{\le N} v) + {\mathbf {P}}_{>N} v\Vert _{L^2 } \le r\}} e^{-\frac{1}{2} E_t( \widetilde{\Psi }_N(t, \tau )({\mathbf {P}}_{\le N} v))} dL_N\bigg \} d\mu _{s, N}^\perp \nonumber \\&= \widehat{Z}_{s, N, r}^{-1} \int _{E_N^\perp } \bigg \{ \int _{E_N} {\mathbf {1}}_{\Psi _N(t, \tau )A} ( \Psi _N(t, \tau ) (v) ) \nonumber \\&\quad \times {\mathbf {1}}_{\{ \Vert \Psi _N(t, \tau ) v\Vert _{L^2 } \le r\}} e^{-\frac{1}{2} E_t( {\mathbf {P}}_{\le N} \Psi _N(t, \tau ) (v))} dL_N\bigg \} d\mu _{s, N}^\perp . \end{aligned}$$

By the bijectivity of $\Psi _N(t, \tau )$, we have ${\mathbf {1}}_{\Psi _N(t, \tau )(A)} ( \Psi _N(t, \tau ) (v) ) = {\mathbf {1}}_{A} ( v )$. We also have the $L^2$-conservation: $\Vert \Psi _N(t, \tau ) ( v)\Vert _{L^2 } = \Vert v\Vert _{L^2 }$ Hence, we have

$$\begin{aligned} \rho _{s, N, r, t} (\Psi _N(t, \tau )(A)) = \widehat{Z}_{s, N, r}^{-1} \int _{L^2} {\mathbf {1}}_{A} ( v ) {\mathbf {1}}_{\{\Vert v\Vert _{L^2 } \le r\}} e^{-\frac{1}{2} E_t( {\mathbf {P}}_{\le N} \Psi _N(t, \tau )( v))} dL_N\otimes d\mu _{s, N}^\perp . \end{aligned}$$

This proves the second equality in (6.28). $\square$

### On the evolution of the truncated measures

In this subsection, we establish a growth estimate on the truncated measure $\rho _{s, N, r, t}$. The key ingredients are the energy estimate (Proposition 6.1), the large deviation estimate (Lemma 6.5), and the change-of-variable formula (Proposition 6.6) from the previous subsections.

#### Lemma 6.8

Let $s > \frac{3}{4}$. There exists $0 \le \beta < 1$ such that, given $r > 0$, there exists $C >0$ such that

6.30 $$\begin{aligned} \frac{d}{dt} \rho _{s, N, r, t}(\Psi _N(t) (A)) \le C p^\beta \big \{ \rho _{s, N, r, t} (\Psi _N(t)(A))\big \}^{1-\frac{1}{p}} \end{aligned}$$

for any $p \ge 2$, any $N \in {\mathbb {N}}$, any $t \in {\mathbb {R}}$, and any measurable set $A \subset L^2({\mathbb {T}})$. Here, $\Psi _N(t) = \Psi _N(t, 0)$ as in (6.4).

As in [69, 71, 72], the main idea of the proof of Lemma 6.8 is to reduce the analysis to that at $t = 0$.

#### Proof

Let $t_0 \in {\mathbb {R}}$. By the definition of $\Psi (t, \tau )$ and Proposition 6.6, we have

$$\begin{aligned} \frac{d}{dt}\rho _{s, N, r, t}&(\Psi _N(t)(A))\bigg |_{t = t_0}\nonumber \\&= \frac{d}{dt}\rho _{s, N, r, t_0+t} \big (\Psi _N(t_0+ t, t_0)(\Psi _N(t_0)(A))\big )\bigg |_{t = 0}\nonumber \\&= \widehat{Z}_{s, N, r}^{-1} \frac{d}{dt} \int _{\Psi _N(t_0) (A)} {\mathbf {1}}_{\{ \Vert v\Vert _{L^2 } \le r\}} e^{-\frac{1}{2} E_{t_0+t}( {\mathbf {P}}_{\le N} \Psi _N(t_0+ t, t_0) (v))} d L_N \otimes d\mu _{s, N}^\perp \bigg |_{t = 0} \nonumber \\&= - \frac{1}{2} \int _{\Psi _N(t_0)( A)} \frac{d}{dt} E_{t_0+t}\big ( {\mathbf {P}}_{\le N} \Psi _N(t_0 + t, t_0) (v)\big )\bigg |_{t = 0} d \rho _{s, N, r, t_0}. \end{aligned}$$

Hence, by Proposition 6.1, Hölder’s inequality, and Lemma 6.5, we have

$$\begin{aligned} \frac{d}{dt}\rho _{s, N, r, t} (\Psi _N(t)(A))\bigg |_{t = t_0}&\le C \Big \Vert \Vert v \Vert _{L^2}^{4+ \theta } \Vert v\Vert _{H^{s - \frac{1}{2} - \varepsilon }}^{2-\theta } \Big \Vert _{L^p(\rho _{s, N, r, t_0})} \big \{ \rho _{s, N, r, t_0}(\Psi _N(t_0) (A))\big \}^{1 - \frac{1}{p}} \nonumber \\&\le C r^{4+\theta } \Big \Vert \Vert v\Vert _{H^{s - \frac{1}{2} - \varepsilon }} \Big \Vert _{L^{(2-\theta )p}(\rho _{s, N, r, t_0})}^{2-\theta } \big \{ \rho _{s, N, r, t_0}(\Psi _N(t_0) (A))\big \}^{1 - \frac{1}{p}} \nonumber \\&\le C_r p^{1- \frac{\theta }{2}} \big \{ \rho _{s, N, r, t_0}(\Psi _N(t_0) (A))\big \}^{1 - \frac{1}{p}} \end{aligned}$$

for some small $\theta > 0$. This proves (6.30) with $\beta = 1- \frac{\theta }{2}$. $\square$

As a corollary to Lemma 6.8, we obtain the following control on the truncated measures $\rho _{s, N, r, t}$.

#### Lemma 6.9

Let $s > \frac{3}{4}$. Then, given $t \in {\mathbb {R}}$, $r > 0$, and $\delta >0$, there exists $C = C(t, r, \delta ) > 0$ such that

$$\begin{aligned} \rho _{s, N, r, t} (\Psi _N(t) (A)) \le C\big \{\rho _{s, N, r, t} ( A) \big \}^{1-\delta } \end{aligned}$$

for any $N \in {\mathbb {N}}$ and any measurable set $A \subset L^2({\mathbb {T}})$.

#### Proof

As in [70], we apply a variant of Yudovich’s argument [73]. From Lemma 6.8, we have

6.31 $$\begin{aligned} \frac{d}{dt}\big \{ \rho _{s, N, r, t}(\Psi _N(t) (A))\big \}^\frac{1}{p} \le C p^{-\alpha } \end{aligned}$$

for any $p \ge 2$, where $\alpha = 1 - \beta > 0$. Integrating (6.31), we have

6.32 $$\begin{aligned} \rho _{s, N, r, t}(\Psi _N(t) (A))&\le \big \{ ( \rho _{s, N, r, t}(A))^\frac{1}{p} + C t p^{-\alpha }\big \}^p \nonumber \\&= \rho _{s, N, r, t}(A) e^{p \log \big \{1 + C t p^{-\alpha } \rho _{s, N, r, t}(A)^{-\frac{1}{p}}\big \}}\nonumber \\&\le \rho _{s, N, r, t}(A) e^{ C t p^{1-\alpha } \rho _{s, N, r, t}(A)^{-\frac{1}{p}}}, \end{aligned}$$

where, in the last inequality, we used the fact that $\log (1+x) \le x$ for $x \ge 0$. By choosing $p = 2 - \log \rho _{s, N, r, t}(A)$ such that

$$\begin{aligned} \rho _{s, N, r, t}(A)^{-\frac{1}{p}} = e^{ 1- \frac{2}{p} } \le e, \end{aligned}$$

it follows from (6.32) that

6.33 $$\begin{aligned} \rho _{s, N, r, t}(\Psi _N(t) (A)) \le \rho _{s, N, r, t}(A) e^{ C e t \{ 2 - \log \rho _{s, N, r, t}(A)\}^{1-\alpha }}. \end{aligned}$$

We claim that, given $\delta > 0$, there exists $C = C(t, \delta , \alpha ) > 0$ such that

6.34 $$\begin{aligned} e^{ C e t \{ 2 - \log \rho \}^{1-\alpha }} \le C(t, \delta , \alpha ) \rho ^{-\delta } \end{aligned}$$

for all $\rho \in [0, 1]$. By rewriting (6.34), it suffices to prove

6.35 $$\begin{aligned} \{ 2 - \log \rho \}^{1-\alpha } \le -\delta \log \rho + \log C(t, \delta , \alpha ) \end{aligned}$$

Clearly, (6.35) holds as $\rho \rightarrow 1-$ by choosing sufficiently large $C(t, \delta , \alpha ) >0$. On the other hand, (6.35) also holds as $\rho \rightarrow 0+$, since $\alpha > 0$. Hence, (6.35) holds for all $\rho \in [0, 1]$ by the continuity of $\log \rho$ and choosing sufficiently large $C(t, \delta , \alpha ) >0$.

Therefore, from (6.33) and (6.34), we conclude that given $\delta > 0$, there exists $C = C(t, r, \delta , \alpha ) > 0$ such that

$$\begin{aligned} \rho _{s, N, r, t} (\Psi _N(t) (A)) \le C(t, r, \delta , \alpha ) \big \{\rho _{s, N, r, t} ( A) \big \}^{1-\delta }. \end{aligned}$$

This completes the proof of Lemma 6.9. $\square$

### Proof of Theorem 1.2

We conclude this section by presenting the proof of Theorem 1.2 for $s > \frac{3}{4}$. Before doing so, we first upgrade Lemma 6.9 to the untruncated measure $\rho _{s, r, t}$.

#### Lemma 6.10

Let $s > \frac{3}{4}$. Then, given $t \in {\mathbb {R}}$, $r > 0$, $R >0$, and $\delta >0$, there exists $C = C(t, r, \delta ) > 0$ such that

6.36 $$\begin{aligned} \rho _{s, r, t} (\Psi (t) (A)) \le C\big \{\rho _{s, r, t} ( A) \big \}^{1-\delta }. \end{aligned}$$

for any measurable set $A \subset L^2({\mathbb {T}})$.

#### Proof

Given $R > 0$, let $B_R$ denote the ball of radius *R* centered at the origin in $L^2({\mathbb {T}})$. We first consider the case when *A* is compact in $L^2$ and $A\subset B_R$ for some $R >0$. It follows from Proposition 6.21 and Corollary 6.3 that, given $\varepsilon , \gamma > 0$, there exists $N_0 = N_0(t, R, \varepsilon , \gamma )\in {\mathbb {N}}$ such that

$$\begin{aligned} \rho _{s, r, t} (\Psi (t) (A))&\le \rho _{s, r, t} (\Psi _N(t) (A+B_\varepsilon )) \le \rho _{s, N, r, t} (\Psi _N(t) (A+B_\varepsilon )) + \gamma \end{aligned}$$

for any $N \ge N_0$. Then, by Lemma 6.9 and Corollary 6.3, we have

6.37 $$\begin{aligned} \rho _{s, r, t} (\Psi (t) (A))&\le C(t, r, \delta ) \big \{\rho _{s, N, r, t} (A+B_\varepsilon ) \big \}^{1-\delta } + \gamma \nonumber \\&\le C(t, r, \delta ) \big \{\rho _{s, r, t} ( A+B_\varepsilon ) \big \}^{1-\delta } + 2 \gamma . \end{aligned}$$

Hence, by taking a limit of (6.37) as $\varepsilon , \gamma \rightarrow 0$ (with the continuity from above of a probability measure), we obtain (6.36) in this case.

Next, let *A* be any measurable set in $L^2$. Then, by the inner regularity of $\rho _{s, r, t}$, there exists a sequence $\{K_j\}_{j \in {\mathbb {N}}}$ of compact sets such that $K_j \subset \Psi (t)( A)$ and

6.38 $$\begin{aligned} \rho _{s, r, t} (\Psi (t) (A)) = \lim _{j \rightarrow \infty } \rho _{s, r, t} (K_j). \end{aligned}$$

By the bijectivity of $\Psi (t, \tau )$, we have

$$\begin{aligned} K_j = \Psi (t, 0) (\Psi (0, t) (K_j))= \Psi (t) (\Psi (0, t) (K_j)). \end{aligned}$$

Note that $\Psi (0, t) (K_j)$ is compact since it is the image of a compact set $K_j$ under the continuous map $\Psi (0, t)$. Moreover, we have $\Psi (0, t) (K_j) \subset \Psi (0, t) \Psi (t) (A) = A$. Then, by (6.36) applied to $\Psi (0, t) (K_j)$, we have

6.39 $$\begin{aligned} \rho _{s, r, t} (K_j)&= \rho _{s, r, t} \big (\Psi (t) (\Psi (0, t) (K_j))\big ) \le C\big \{\rho _{s, r, t} ( \Psi (0, t) (K_j) ) \big \}^{1-\delta }\nonumber \\&\le C\big \{\rho _{s, r, t} ( A) \big \}^{1-\delta }. \end{aligned}$$

By taking a limit as $j \rightarrow \infty$, we obtain (6.36) from (6.38) and (6.39). $\square$

Finally, we present the proof of Theorem 1.2.

#### Proof of Theorem 1.2

As in Sect. 5, it follows from Lemmas 4.1, 4.4, and 4.5 that it suffices to prove that $\mu _s$ is quasi-invariant under $\Psi (t)$, i.e. the dynamics of (3.6).

Fix $t \in {\mathbb {R}}$. Let $A \subset L^2({\mathbb {T}})$ be a measurable set such that $\mu _s(A) = 0$. Then, for any $r > 0$, we have

$$\begin{aligned} \mu _{s, r}(A) = 0. \end{aligned}$$

By the mutual absolute continuity of $\mu _{s, r}$ and $\rho _{s, r, t}$, we obtain

$$\begin{aligned} \rho _{s, r, t}(A) = 0 \end{aligned}$$

for any $r > 0$. Then, by Lemma 6.10, we have

$$\begin{aligned} \rho _{s, r, t}(\Psi (t) (A)) = 0. \end{aligned}$$

By invoking the mutual absolute continuity of $\mu _{s, r}$ and $\rho _{s, r, t}$ once again, we have

$$\begin{aligned} \mu _{s, r}(\Psi (t) (A)) = 0. \end{aligned}$$

Then, the dominated convergence theorem yields

$$\begin{aligned} \mu _{s}\big (\Psi (t) (A)\big ) = \lim _{r \rightarrow \infty } \mu _{s, r}\big (\Psi (t) (A)\big ) = 0. \end{aligned}$$

This completes the proof of Theorem 1.2. $\square$

## Appendix A. On the Cauchy problem (1.1)

In this appendix, we discuss the well-posedness issue for the Cauchy problem (1.1). In particular, we prove global well-posedness of (1.1) in $L^2({\mathbb {T}})$ and ill-posedness below $L^2({\mathbb {T}})$.

### Well-posedness in $L^2({\mathbb {T}})$

We say *u* is a solution to (1.1) if *u* satisfies the following Duhamel formulation

$$\begin{aligned} u (t) = S(t) u_0 \mp i \int _0^t S(t - t') |u|^2 u(t') dt', \end{aligned}$$

where $S(t) = e^{-i t\partial _x^4}$. The main result of this section is the following local well-posedness of (1.1).

#### Proposition 6.11

Let $s \ge 0$. Then, given $u_0\in H^s({\mathbb {T}})$, there exist $T = T(\Vert u_0\Vert _{L^2}) > 0$ and a unique solution $u \in C([-T, T]; H^s)$ to (1.1) with $u|_{t = 0} = u_0$. Moreover, we have

A.1 $$\begin{aligned} \sup _{t \in [-T, T]}\Vert u(t) \Vert _{H^s}&\le C \Vert u_0 \Vert _{H^s}. \end{aligned}$$

See Remark 6.12 below for the precise uniqueness statement. Once we prove Proposition 6.11, global well-posedness (Proposition 1.1) follows from the conservation of mass (1.4). We prove Proposition 6.11 via the Fourier restriction norm method [7]. While the argument is standard, we present the details of the proof for the sake of completeness.

Given $s, b \in {\mathbb {R}}$, define $X^{s, b}$ as the completion of ${\mathcal {S}}({\mathbb {T}}\times {\mathbb {R}})$ under the following norm:

A.2 $$\begin{aligned} \Vert u\Vert _{X^{s, b}({\mathbb {T}}\times {\mathbb {R}})} = \Vert \langle n \rangle ^s \langle \tau + n^4 \rangle ^b \widehat{u}(n, \tau )\Vert _{\ell ^2_nL^2_\tau }. \end{aligned}$$

Given a time interval $I \subset {\mathbb {R}}$, we define the local-in-time version $X^{s, b}_I$ restricted to the time interval *I* by setting

$$\begin{aligned} \Vert u \Vert _{X^{s, b}(I)} = \inf \big \{ \Vert \widetilde{u} \Vert _{X^{s, b}} : \, \widetilde{u}|_I = u\big \}. \end{aligned}$$

#### Remark 6.12

When $s > \frac{1}{2}$, the uniqueness statement in Proposition 6.11 holds in $C([-T, T]; H^s)$. When $s \in [0, \frac{1}{2}]$, the uniqueness holds only within a ball in $C([-T, T]; H^s) \cap X^{s, b}([-T, T])$ for some $b > \frac{1}{2}$.^8^

Before presenting the proof of Proposition 6.11, we first go over preliminary lemmas. Let $\eta \in C^\infty _c({\mathbb {R}})$ be a smooth cut off function such that $\eta (t) \equiv 1$ for $|t|\le 1$ and $\eta (t) \equiv 0$ for $|t|\ge 2$. Given $T>0$, set $\eta _{_T}(t) = \eta (T^{-1} t)$. Then, we have the following basic linear estimates. See [7, 34, 43, 64]

#### Lemma 6.13

Let $$s \in {\mathbb {R}}$$.

(i) For any $b \in {\mathbb {R}}$, we have

$$\begin{aligned} \Vert \eta (t) S(t) u_0 \Vert _{X^{s, b}} \le C_b \Vert u_0\Vert _{H^{s}}. \end{aligned}$$

(ii) Let $- \frac{1}{2} < b' \le 0 \le b \le b'+1$. Then, for $T \le 1$, we have

$$\begin{aligned} \bigg \Vert \eta _{_T} (t) \int _0^t S(t-t')F(t') dt'\bigg \Vert _{X^{s, b}} \le C_{b, b'} T^{1-b+b'} \Vert F\Vert _{X^{s, b'}}. \end{aligned}$$

Next, we state the $L^4$-Strichartz estimate.

#### Lemma 6.14

The following estimate holds:

A.3 $$\begin{aligned} \Vert u \Vert _{L^4({\mathbb {T}}\times {\mathbb {R}})} \lesssim \Vert u \Vert _{X^{0, \frac{5}{16}}}. \end{aligned}$$

Note that the value $b = \frac{5}{16}$ in (A.3) is sharp in the sense that the estimate (A.3) fails for $b < \frac{5}{16}$.^9^

#### Proof

We closely follow the argument for the $L^4$-Strichartz estimate for the usual (second order) Schrödinger equation presented in [64]. Given dyadic $M \ge 1$, let $u_M$ be the restriction of *u* onto the modulation size $\langle \tau + n^4 \rangle \sim M$. Then, it suffices to show that there exists $\varepsilon > 0$ such that

A.4 $$\begin{aligned} \Vert u_M u_{2^m M} \Vert _{L^2_{x, t}} \lesssim 2^{-\varepsilon m } M^\frac{5}{16}\Vert u_M\Vert _{L^2_{x, t}} ( 2^mM)^\frac{5}{16}\Vert u_{2^mM}\Vert _{L^2_{x, t}} \end{aligned}$$

for any $M \in {\mathbb {N}}$ and $m \in {\mathbb {N}}\cup \{0\}$.

Indeed, assuming (A.4), by Cauchy-Schwarz inequality, we have

$$\begin{aligned} \Vert u \Vert _{L^4({\mathbb {T}}\times {\mathbb {R}})}^2&\lesssim \sum _{M } \sum _{m \ge 0} \Vert u_M u_{2^m M} \Vert _{L^2_{x, t}}\nonumber \\&\lesssim \sum _{M } \sum _{m \ge 0} 2^{-\varepsilon m } M^\frac{5}{16}\Vert u_M\Vert _{L^2_{x, t}} ( 2^mM)^\frac{5}{16}\Vert u_{2^mM}\Vert _{L^2_{x, t}} \nonumber \\&\lesssim \sum _{m \ge 0} 2^{-\varepsilon m } \bigg ( \sum _{M } M^\frac{5}{8}\Vert u_M\Vert _{L^2_{x, t}}^2\bigg )^\frac{1}{2} \bigg ( \sum _{M }( 2^mM)^\frac{5}{8}\Vert u_{2^mM}\Vert _{L^2_{x, t}}^2\bigg )^\frac{1}{2}\\&\lesssim \Vert u \Vert _{X^{0, \frac{5}{16}}}^2. \end{aligned}$$

This proves (A.3).

Hence, it remains to prove (A.4). By Plancherel’s identity and Hölder’s inequality, we have

A.5 $$\begin{aligned} \text {LHS of }(A.4)&= \bigg \Vert \sum _{n = n_1 + n_2} \mathop {{{\mathrm{\int }}}}\limits _{\tau = \tau _1 + \tau _2} \widehat{u_M}(n_1, \tau _1) \widehat{u_{2^m M}} (n_2, \tau _2) d\tau _1\bigg \Vert _{\ell ^2_n L^2_\tau }\nonumber \\&\le \sup _{n, \tau } A(n, \tau )^\frac{1}{2} \cdot \Vert u_M\Vert _{L^2_{x, t}} \Vert u_{2^mM}\Vert _{L^2_{x, t}} , \end{aligned}$$

where $A(n, \tau )$ is defined by

$$\begin{aligned} A(n, \tau ) = \sum _{n = n_1 + n_2} \mathop {{{\mathrm{\int }}}}\limits _{\tau = \tau _1 + \tau _2} {\mathbf {1}}_{\tau _1 +n_1^4 = O(M), \, \tau _2 +n_2^4 = O(2^mM)}\, d\tau _1 . \end{aligned}$$

Integrating in $\tau _1$, we have

A.6 $$\begin{aligned} A(n, \tau ) \lesssim M \sum _{n = n_1 + n_2} {\mathbf {1}}_{\tau = - n_1^4 - n_2^4 + O(2^mM)}. \end{aligned}$$

Under $n = n_1 + n_2$ and $\tau = - n_1^4 - n_2^4 + O(2^mM)$, we have

$$\begin{aligned} \big ( (n_1 - n_2)^2 + 3 n^2 \big )^2 = 8 (n_1^4 + n_2^4) + 8 n^4 = -8 \tau + 8 n^4 + O(2^mM). \end{aligned}$$

This implies that $(n_1 - n_2)^2 + 3 n^2$ belongs to at most two intervals of size $O(2^\frac{m}{2}M^\frac{1}{2})$, i.e.

$$\begin{aligned} (n_1 - n_2)^2 + 3 n^2 = C_{j, \tau , n} + O(2^\frac{m}{2}M^\frac{1}{2}) \end{aligned}$$

for some $C_{j, \tau , n}$, $j = 1, 2$. This, in turn, implies that $n_1-n_2$ belongs to at most four intervals of size $O(2^\frac{m}{4}M^\frac{1}{4})$. Hence, from (A.6), we have

A.7 $$\begin{aligned} A(n, \tau ) ^\frac{1}{2} \lesssim 2^\frac{m}{8}M^\frac{5}{8} \le 2^{-\frac{3}{16}m}M^{\frac{5}{16}} (2^m M)^{\frac{5}{16}}. \end{aligned}$$

Finally, (A.4) follows from (A.5) and (A.7). $\square$

Now, we are ready to prove Proposition 6.11.

#### Proof of Proposition 6.11

Let $u_0 \in L^2({\mathbb {T}})$. Given $0< T \le 1$, let

A.8 $$\begin{aligned} \Gamma (u) (t) = \Gamma _{u_0}(u) (t) : = \eta (t) S(t) u_0 \mp i \eta _{_T}(t) \int _0^t S(t - t') |u|^2 u(t') dt'. \end{aligned}$$

Let $b >\frac{1}{2}$ and small $\delta > 0$. Then, from Lemma 6.13, a duality argument, Hölder’s inequality, and Lemma 6.14, we have

A.9 $$\begin{aligned} \Vert \Gamma (u) \Vert _{X^{0,b}}&\lesssim \Vert u_0 \Vert _{L^2} + T^\delta \big \Vert |u|^2 u \big \Vert _{X^{0, b - 1+\delta }} \nonumber \\&= \Vert u_0 \Vert _{L^2} + T^\delta \sup _{\Vert v\Vert _{X^{0, 1-b-\delta } = 1 }} \bigg | \int |u|^2 u \cdot v \, dx dt\bigg |\nonumber \\&\le \Vert u_0 \Vert _{L^2} + T^\delta \sup _{\Vert v\Vert _{X^{0, 1-b-\delta } = 1 }} \Vert u\Vert ^3_{L^4_{x, t}} \Vert v\Vert _{L^4_{x, t}}\nonumber \\&\lesssim \Vert u_0 \Vert _{L^2} + T^\delta \Vert u\Vert ^3_{X^{0, \frac{5}{16}}}, \end{aligned}$$

as long as $b \le \frac{11}{16} - \delta$. Similarly, we have

A.10 $$\begin{aligned} \Vert \Gamma (u) - \Gamma (v) \Vert _{X^{0,b}} \lesssim T^\delta \big ( \Vert u\Vert ^2_{X^{0, b}} + \Vert v\Vert ^2_{X^{0, b}}\big ) \Vert u - v\Vert _{X^{0, b}}. \end{aligned}$$

Hence, it follows from (A.9) and (A.10) that $\Gamma$ is a contraction on some ball in $X^{0, b}$ as long as $T = T(\Vert u_0\Vert _{L^2}) > 0$ is sufficiently small.

Now, suppose that $u_0 \in H^s({\mathbb {T}})$ for some $s > 0$. Then, proceeding as in (A.9) with $T = T(\Vert u_0\Vert _{L^2}) > 0$ as above, we have

A.11 $$\begin{aligned} \Vert \Gamma (u) \Vert _{X^{s, b}}&\lesssim \Vert u_0 \Vert _{H^s} + T^\delta \Vert u\Vert ^2_{X^{0, b}} \Vert u\Vert _{X^{s, b}} \lesssim \Vert u_0 \Vert _{H^s} + T^\delta \Vert u_0\Vert _{L^2}^2 \Vert u\Vert _{X^{s, b}}, \end{aligned}$$

yielding (A.1). A similar argument yields local Lipschitz dependence of the solution map on $H^s({\mathbb {T}})$. This completes the proof of Proposition 6.11. $\square$

#### Remark 6.15

When $s = 0$, the conservation of mass yields $\Vert u(t) \Vert _{L^2} = \Vert u_0 \Vert _{L^2}$ for all $t \in {\mathbb {R}}$.

Now, suppose that $s>0$. Then, by iterating (A.1) along with the mass conservation, we conclude that there exists $\theta > 0$ such that the following growth estimate on the $H^s$-norm holds:

A.12 $$\begin{aligned} \sup _{t \in [0, \tau ]}\Vert u(t) \Vert _{H^s}&\le C^{K^\theta \tau } \Vert u_0 \Vert _{H^s} \end{aligned}$$

for any $\tau > 0$ and all $u_0 \in H^s({\mathbb {T}})$ with $\Vert u_0 \Vert _{L^2}\le K$.

### Ill-posedness below $L^2({\mathbb {T}})$

In the following, we briefly discuss the ill-posedness of (1.1) below $L^2({\mathbb {T}})$. We first present the following failure of uniform continuity of the solution map on bounded sets below $L^2({\mathbb {T}})$.

#### Lemma 6.16

Let $s < 0$. There exist two sequences $\{u_{0, n}\}_{n \in {\mathbb {N}}}$ and $\{\widetilde{u}_{0, n}\}_{n \in {\mathbb {N}}}$ in $H^\infty ({\mathbb {T}})$ such that

(i) $u_{0, n}$ and $\widetilde{u}_{0, n}$ are uniformly bounded in $H^s({\mathbb {T}})$,
(ii) $$\displaystyle \lim \limits _{n \rightarrow \infty } \Vert u_{0, n}- \widetilde{u}_{0, n}\Vert _{H^s} = 0$$,
(iii) Let $u_n$ and $\widetilde{u}_n$ be the solutions to (1.1) with initial data $u_n|_{t = 0} =u_{0, n}$ and $\widetilde{u}_n|_{t = 0} =\widetilde{u}_{0, n}$, respectively. Then, there exists $c > 0$ such that
  $$\begin{aligned} \liminf _{n \rightarrow \infty } \Vert u_n - \widetilde{u}_n\Vert _{L^\infty ([-T, T]; H^s)} \ge c > 0 \end{aligned}$$
  for any $$T > 0$$.

Lemma 6.16 exhibits a “mild” ill-posedness result for $s< 0$. The proof of Lemma 6.16 closely follows the argument in Burq–Gérard–Tzvetkov [13] and Christ–Colliander–Tao [20].

#### Proof

Given $N \in {\mathbb {N}}$ and $a \in {\mathbb {C}}$, define $u^{(N, a)}$ by

$$\begin{aligned} u^{(N, a)}(x, t) = N^{-s} a e^{i(Nx - N^4 t \mp N^{-2s} |a|^2 t)}. \end{aligned}$$

Then, it is easy to see that $u^{(N, a)}$ is a smooth global solution to (1.1).

Given $n \in {\mathbb {N}}$, let $u_{0, n} = u^{(N_n, 1)}(0)$ and $\widetilde{u}_{0, n} = u^{(N_n, 1 + \frac{1}{n})}(0)$, where $N_n \in {\mathbb {N}}$ is to be chosen later. Then, we have

A.13 $$\begin{aligned} \Vert u_{0, n}\Vert _{H^s} , \Vert \widetilde{u}_{0, n}\Vert _{H^s} \lesssim 1 \end{aligned}$$

uniformly in $n \in {\mathbb {N}}$. Moreover, we have

A.14 $$\begin{aligned} \Vert u_{0, n} - \widetilde{u}_{0, n}\Vert _{H^s} \sim \frac{1}{n}. \end{aligned}$$

Note that (A.13) and (A.14) hold independently of a choice of $N_n \in {\mathbb {N}}$.

Let $u_n$ and $\widetilde{u}_n$ be the solutions to (1.1) with initial data $u_n|_{t = 0} =u_{0, n}$ and $\widetilde{u}_n|_{t = 0} =\widetilde{u}_{0, n}$, respectively. Namely, $u_{ n} = u^{(N_n, 1)}$ and $\widetilde{u}_{n} = u^{(N_n, 1 + \frac{1}{n})}$. Given $n \in {\mathbb {N}}$, define $t_n > 0$ by

$$\begin{aligned} t_n = \frac{\pi N_n^{2s}}{ \big (1+\tfrac{1}{n}\big )^2 - 1} . \end{aligned}$$

Since $s < 0$, we can choose $N_n \in {\mathbb {N}}$ sufficiently large such that $t_n \le \frac{1}{n}$. Then, we have

A.15 $$\begin{aligned} \Vert u_{n}(t_n) - \widetilde{u}_{n}(t_n)\Vert _{H^s} = \Big | e^{\mp i N_n ^{-2s}\{1 - (1+\frac{1}{n})^2\} t_n} - \big (1 + \tfrac{1}{n}\big )\Big | = 2 + \tfrac{1}{n} \ge 2. \end{aligned}$$

Noting that $t_n \rightarrow 0$ as $n \rightarrow \infty$, Lemma 6.16 follows from (A.13), (A.14), and (A.15). $\square$

#### Remark 6.17

The cubic NLS (1.7) enjoys the Galilean symmetry, which preserves the $L^2$-norm. Namely, $L^2$ is critical with respect to the Galilean symmetry. Indeed, the cubic NLS is known to be ill-posed below $L^2({\mathbb {T}})$. See [13, 20, 21, 38, 51].

As for the fourth order NLS (1.1), there seems to be no Galilean symmetry^10^ and it is not clear why the regularity $s = 0$ plays a role as a critical value.

#### Remark 6.18

(non-existence of solutions below $L^2({\mathbb {T}})$) The mild ill-posedness of (1.1) stated in Lemma 6.16 can be updated to the following strong form of ill-posedness of (1.1) below $L^2({\mathbb {T}})$. Roughly speaking, if $u_0 \notin L^2({\mathbb {T}})$, then there is no weak solution to (1.1). More precisely, there exists $s_0 < 0$ such that, for $s_0< s < 0$ and any $T>0$, there exists no weak solution $u \in C([-T, T];H^s({\mathbb {T}}))$ to NLS (1.1) such that

(i) $$u|_{t = 0} = u_0 \in H^s({\mathbb {T}}){\setminus } L^2({\mathbb {T}})$$
(ii) There exist smooth solutions $\{u_n\}_{n\in {\mathbb {N}}}$ to (1.1) such that $u_n \rightarrow u$ in $C([-T, T];H^s({\mathbb {T}}))$ as $n \rightarrow \infty$.

Note that this is one of the strongest forms of ill-posedness.

In the following, we present a sketch of the argument. See [38, 59] for details. Namely, first use the short time Fourier restriction norm method and establish an a priori bound in $H^s$, $s< 0$, to the renormalized Eq. (3.3). Here, the main observation is that Lemma 3.1 guarantees that the a priori bound for the renormalized cubic NLS also holds for the renormalized fourth order NLS (3.3).^11^ This allows us to prove an existence result for (3.3) in $H^s({\mathbb {T}})$, $s_0< s < 0$, for some $s_0 < 0$. Recall that if *u* is a smooth solution to (1.1), then $\widetilde{u} = {\mathcal {G}}[u]$ is a smooth solution to (3.3).

Now, let $u_0 \in H^s({\mathbb {T}}){\setminus } L^2({\mathbb {T}})$, $s \in (s_0, 0)$ and let $\{u_{0, n}\}_{n \in {\mathbb {N}}} \subset L^2({\mathbb {T}})$ such that $u_{0, n} \rightarrow u_0$ in $H^s({\mathbb {T}})$ as $n \rightarrow \infty$. Let $u_n$ denote the unique (global) solution to (1.1) with $u_n|_{t = 0} = u_{0, n}$ and let $\widetilde{u}_n = {\mathcal {G}} [u_n]$. Then, from the a priori bound, there exists $T = T(\Vert u_0\Vert _{H^s})>0$ such that (i) $\{\widetilde{u}_n\}_{n \in {\mathbb {N}}}$ is bounded in $C([-T, T]; H^s)$ and (ii) $\widetilde{u}_n$ converges to some $\widetilde{u}$ in $C([-T, T]; H^s)$. Moreover, $\widetilde{u}$ is a solution to (3.3). In particular, $\widetilde{u}(0) = u_0$. On the other hand, in view of $\Vert u_n (0)\Vert _{L^2} \rightarrow \infty$ as $n \rightarrow \infty$, we have faster and faster phase oscillations in (3.2), as $n \rightarrow \infty$. Hence, $\widetilde{u}_n = {\mathcal {G}}[ u_n]$ converges to 0 in ${\mathcal {D}}'({\mathbb {T}}\times [-T, T])$. In particular, this implies $\widetilde{u}(0) = 0$. This is clearly a contradiction since $\widetilde{u}(0) = u_0 \notin L^2({\mathbb {T}})$.

## Appendix B. On the approximation property of the truncated dynamics

In this appendix, we perform further analysis on the Eq. (3.6) and its truncated approximation (6.1) and establish a certain approximation property. See Proposition 6.21 below.

Given $N \in {\mathbb {N}}$, we first consider the following approximation to (1.1):

A.16 $$\begin{aligned} {\left\{ \begin{array}{ll} i \partial _tu_N = \partial _x^4 u_N - {\mathbf {P}}_{\le N} (|{\mathbf {P}}_{\le N}u_N|^{2}{\mathbf {P}}_{\le N}u_N) \\ u_N|_{t = 0} = u_0. \end{array}\right. } \end{aligned}$$

We first study the approximation property of (A.16) to (1.1). By a slight modification of the proof of Proposition 6.11, it is easy to see that (A.16) is globally well-posed in $L^2({\mathbb {T}})$.

Let $$\Phi (t)$$ and $$\Phi _N(t)$$ be the solution maps to (1.1) and (A.16), respectively. Given $$R > 0$$, let $$B_R $$ be the ball of radius *R* centered at the origin in $$L^2({\mathbb {T}})$$. Let $$\tau > 0$$. By iterating the local-in-time argument (see (A.11)), we have the following uniform estimate:

A.17 $$\begin{aligned} \sup _{N\in {\mathbb {N}}\cup \{\infty \}} \sup _{u_0 \in B_R} \Vert \Phi _N(t)(u_0) \Vert _{X^{0, b}([0, \tau ])} \le C(\tau , R) \end{aligned}$$

for some $b > \frac{1}{2}$, with the understanding that $\Phi _\infty (t) = \Phi (t)$. Then, from Lemma 6.14, we obtain

A.18 $$\begin{aligned} \sup _{N\in {\mathbb {N}}\cup \{\infty \}} \sup _{u_0 \in B_R} \Vert \Phi _N(t)(u_0) \Vert _{L^4_t([0, \tau ]; L^4_x)} \le C(\tau , R). \end{aligned}$$

### Lemma 6.19

Given $R > 0$, let $A \subset B_R$ be a compact subset in $L^2({\mathbb {T}})$. Given $\tau > 0$ and $\varepsilon >0$, there exists $N_0 \in {\mathbb {N}}$ such that we have

A.19 $$\begin{aligned} \Vert {\mathbf {P}}_{>N} \Phi (t)(u_0)\Vert _{L^4([0, \tau ]; L^4_x)} < \varepsilon \end{aligned}$$

for all $u_0 \in A$ and $N \ge N_0$.

### Proof

By the continuity of the map: $u_0 \in L^2 \mapsto \Phi (t) u_0 \in L^4([0, \tau ]; L^4_x)$ and the compactness of *K*, we see that $\Phi (t) K$ is compact in $L^4([0, \tau ]; L^4_x)$. Hence, there exists a finite index set ${\mathcal {J}}$ and $\{u_{0, j}\}_{j \in {\mathcal {J}}} \subset K$ such that, given $u_0 \in K$, we have

A.20 $$\begin{aligned} \Vert \Phi (t) (u_0) - \Phi (t) (u_{0, j})\Vert _{L^4([0, \tau ]; L^4_x)} <\frac{\varepsilon }{2} \end{aligned}$$

for some $j \in {\mathcal {J}}$. It follows from (A.18) and the dominated convergence theorem that given $j \in {\mathcal {J}}$, there exists $N_j \in {\mathbb {N}}$ such that

A.21 $$\begin{aligned} \Vert {\mathbf {P}}_{>N} \Phi (t)(u_{0, j})\Vert _{L^4([0, \tau ]; L^4_x)} < \frac{\varepsilon }{2} \end{aligned}$$

for all $N \ge N_j$. Hence, by setting $N_0 = \max _{j \in {\mathcal {J}}} N_j$, (A.19) follows from (A.20) and (A.21). $\square$

We first establish the following approximation property of (A.16) to (1.1).

### Lemma 6.20

Given $R > 0$, let $A \subset B_R$ be a compact set in $L^2({\mathbb {T}})$. Then, for any $\tau > 0$ and $\varepsilon > 0$, there exists $N_0 \in {\mathbb {N}}$ such that

A.22 $$\begin{aligned} \Vert \Phi (t)(u_{0}) - \Phi _N(t)(u_{0})\Vert _{L^\infty _t([0, \tau ]; L^2_x)} < \varepsilon . \end{aligned}$$

for all $u_0 \in A$ and $N \ge N_0$.

### Proof

Given $u_0 \in A$, let $w_N = u - {\mathbf {P}}_{\le N} u_N = \Phi (t) (u_0) -{\mathbf {P}}_{\le N} \Phi _N(t)(u_0)$. Then, $w_N$ satisfies

$$\begin{aligned} w_N(t) = - i \int _0^t S(t - t') {\mathcal {Q}}(u, {\mathbf {P}}_{\le N} u_N)(t') dt'. \end{aligned}$$

where ${\mathcal {Q}}(u, {\mathbf {P}}_{\le N}u_N)$ is defined by

$$\begin{aligned} {\mathcal {Q}}(u, {\mathbf {P}}_{\le N}u_N)&= {\mathbf {P}}_{\le N }\big ( |u|^2 u - |{\mathbf {P}}_{\le N} u|^2 {\mathbf {P}}_{\le N} u\big ) + {\mathbf {P}}_{> N } (|u|^2 u). \end{aligned}$$

Given $T> 0$, let $\widetilde{u}$ and $\widetilde{u}_N$ be extensions of $u |_{[0, T]}$ and ${\mathbf {P}}_{\le N} u_N |_{[0, T]}$ onto ${\mathbb {R}}$. Then, defining $\widetilde{w}_N$ by

$$\begin{aligned} \widetilde{w}_N(t) = - i \eta _{_T}(t) \int _0^t S(t - t') {\mathcal {Q}}(\widetilde{u}, \widetilde{u}_N)(t') dt', \end{aligned}$$

we see that $\widetilde{w}_N$ is an extension of $w_N|_{[0, T]}$ onto ${\mathbb {R}}$. Given small $\delta > 0$, let $\frac{1}{2} < b \le \frac{11}{16}-\delta$ as in the proof of Proposition 6.11. Then, by proceeding as in (A.9), we have

A.23 $$\begin{aligned} \Vert w_N \Vert _{X^{0, b} ([0, T])}&\le \Vert \widetilde{w}_N \Vert _{X^{0, b} ({\mathbb {T}}\times {\mathbb {R}})}\nonumber \\&\le C T^\delta \big ( \Vert \widetilde{u} \Vert _{X^{0, \frac{5}{16}}}^2 + \Vert \widetilde{u}_N \Vert _{X^{0, \frac{5}{16}}}^2\big ) \Vert \widetilde{u} - \widetilde{u}_N \Vert _{X^{0, \frac{5}{16}}} \nonumber \\&\quad + C\Vert \widetilde{u} \Vert _{L^4_{x, t}}^2 \Vert {\mathbf {P}}_{> \frac{N}{3}} \widetilde{u} \Vert _{L^4_{x, t}} \end{aligned}$$

for $T = T(R) > 0$ sufficiently small. Noting that (A.23) holds for any extensions $\widetilde{u}$ and $\widetilde{u}_N$ and that $\widetilde{u} - \widetilde{u}_N$ is an extension of $w_N|_{[0, T]}$, we obtain

A.24 $$\begin{aligned} \Vert w_N \Vert _{X^{0, b} ([0, T])}&\le C T^\delta \big ( \Vert u \Vert _{X^{0, \frac{5}{16}}([0, T])}^2 + \Vert u_N \Vert _{X^{0, \frac{5}{16}}([0, T])}^2\big ) \Vert w_N \Vert _{X^{0, \frac{5}{16}}([0, T])} \nonumber \\&\quad + C\Vert u \Vert _{L^4_t([0, T]; L^4_x)}^2 \Vert {\mathbf {P}}_{> \frac{N}{3}} u \Vert _{L^4_t([0, T]; L^4_x)}. \end{aligned}$$

By making $T = T(\tau , R)> 0$ sufficiently small, it follows from (A.24) with (A.17), that

$$\begin{aligned} \Vert w_N \Vert _{L^\infty ([0, T]; L^2)}&\le C \Vert w_N \Vert _{X^{0, b} ([0, T])} \le C\Vert u \Vert _{L^4([0, T]; L^4_x)}^2 \Vert {\mathbf {P}}_{> \frac{N}{3}} u \Vert _{L^4_t([0, T]; L^4_x)}. \end{aligned}$$

Hence, by Lemma 6.19 with (A.18),

$$\begin{aligned} \Vert w_N \Vert _{L^\infty ([0, T]; L^2)} = o_N(1) \end{aligned}$$

as $N \rightarrow \infty$, uniformly in $u_0 \in A$.

By repeating the argument, we obtain

$$\begin{aligned} \Vert w_N \Vert _{X^{0, b} ([T, 2T])}&\le o_N(1) + C T^\delta \big ( \Vert u \Vert _{X^{0, \frac{5}{16}}([T, 2T])}^2 + \Vert u_N \Vert _{X^{0, \frac{5}{16}}([T, 2T])}^2\big ) \Vert w_N \Vert _{X^{0, \frac{5}{16}}([T, 2T])} \nonumber \\&\quad + C\Vert u \Vert _{L^4([T, 2T]; L^4_x)}^2 \Vert {\mathbf {P}}_{> \frac{N}{3}} u \Vert _{L^4([T, 2T]; L^4_x)}. \end{aligned}$$

As before, this in turn implies

$$\begin{aligned} \Vert w_N \Vert _{L^\infty _t([T, 2T]; L^2_x)} = o_N(1) \end{aligned}$$

as $N \rightarrow \infty$, uniformly in $u_0 \in A$. By arguing iteratively on time intervals of length *T*, we can cover the whole time interval $[0, \tau ]$ and we conclude that there exists $N_1 = N_1(\tau , \varepsilon , R) \in {\mathbb {N}}$ such that

A.25 $$\begin{aligned} \Vert \Phi (t) (u_0) -{\mathbf {P}}_{\le N} \Phi _N(t)(u_0) \Vert _{L_t^\infty ([0, \tau ]; L^2_x)} < \frac{\varepsilon }{2} \end{aligned}$$

for all $u_0 \in A$ and $N \ge N_1$.

It remains to control ${\mathbf {P}}_{> N} \Phi _N(t)(u_0)$. Recall that the solution map $\Phi _N(t)$ to (A.16) is locally uniformly continuous. Moreover, it follows from a slight modification of the proof of Proposition 6.11 that the modulus of continuity is uniform in $N \in {\mathbb {N}}$. Hence, for any $\varepsilon > 0$, there exists $\delta > 0$ such that if $u_0, u_1 \in B_R$ satisfies $\Vert u_0 - u_1\Vert _{L^2} < \delta$, then we have

$$\begin{aligned} \Vert \Phi _N(t) (u_0) - \Phi _N(t) (u_1)\Vert _{L^\infty _t([0, \tau ]; L^2_x)} < \frac{\varepsilon }{4} \end{aligned}$$

for all $N \in {\mathbb {N}}$. By the compactness of *A*, we can cover *A* by finitely many ball of radius $\delta$ centered at $u_{0, j}$, $j = 1, \ldots , J$ for some $J < \infty$ such that, given $u_0 \in A$, there exists $j \in \{1, \ldots , J\}$ such that

A.26 $$\begin{aligned} \Vert \Phi _N(t) (u_0) - \Phi _N(t) (u_{0, j})\Vert _{L^\infty _t([0, \tau ]; L^2_x)} < \frac{\varepsilon }{4} \end{aligned}$$

for all $$N \in {\mathbb {N}}$$.

Noting that ${\mathbf {P}}_{> N} \Phi _N(t)(u_{0, j}) = S(t) {\mathbf {P}}_{> N} u_{0, j}$, there exists $N_2 \in {\mathbb {N}}$ such that

A.27 $$\begin{aligned} \Vert {\mathbf {P}}_{> N} \Phi _N(t)(u_{0, j}) \Vert _{L^\infty _t([0, \tau ]; L^2_x)} = \Vert S(t) {\mathbf {P}}_{> N} u_{0, j}\Vert _{L^\infty _t([0, \tau ]; L^2_x)} = \Vert {\mathbf {P}}_{> N} u_{0, j}\Vert _{L^2_x} < \frac{\varepsilon }{4} \end{aligned}$$

for all $N \ge N_2$ and $j = 1, \ldots , J$.

Therefore, the desired estimate (A.22) follows from (A.25), (A.26), and (A.27).

$$\square $$

Recall that, if *u* is a solution to (1.1), then $v(t) = S(-t) {\mathcal {G}}[u](t)$ is a solution to (3.6), where the gauge transformation ${\mathcal {G}}$ is defined in (3.2). Noting that the truncated $L^2$-norm $\int |{\mathbf {P}}_{\le N} u|^2dx$ is conserved for (A.16), define ${\mathcal {G}}_N$ by

A.28

for a solution *u* to (A.16). Then, letting

$$\begin{aligned} v = S(-t) {\mathcal {G}}_N[u](t), \end{aligned}$$

we see that *v* is a solution to (6.1). Recalling that $\Psi (t)= \Psi (t, 0)$ and $\Psi _N(t)= \Psi _N(t, 0)$ represent the solution maps to (3.6) and (6.1), respectively, we have

A.29 $$\begin{aligned} \Psi (t) (u_0) = S(-t) \circ {\mathcal {G}}\circ \Phi (t)(u_0) \qquad \text {and} \qquad \Psi _N(t) (u_0) = S(-t)\circ {\mathcal {G}}_N\circ \Phi _N(t)(u_0). \end{aligned}$$

We conclude this appendix by establishing the following approximation property of (6.1) to (3.6). Lemma 6.20 and (A.29) play an important role.

### Proposition 6.21

Given $R>0$, let $A \subset B_R$ be a compact set in $L^2({\mathbb {T}})$. Fix $t \in {\mathbb {R}}$. Then, for any $\varepsilon > 0$, there exists $N_0 = N_0(t, R, \varepsilon ) \in {\mathbb {N}}$ such that we have

A.30 $$\begin{aligned} \Psi (t) (A)\subset \Psi _N(t) (A + B_\varepsilon ) \end{aligned}$$

for all $$N \ge N_0$$.

### Proof

By writing $\Psi (t)(A)$ as

$$\begin{aligned} \Psi (t) (A) = \Psi _N(t)\big ( \Psi _N(0, t) \Psi (t) (A)\big ), \end{aligned}$$

it suffices to show $\Psi _N(0, t) \Psi (t) (A) \subset A + B_\varepsilon$. Given $w_N \in \Psi _N(0, t) \Psi (t) (A)$, we have $w_N = \Psi _N(0, t) \Psi (t) (u_0)$ for $u_0 \in A$. Thus, we can rewrite $w_N$ as $w_N = u_0+ z_N$, where

$$\begin{aligned} z_N : = \Psi _N(0, t)\big ( \Psi (t)( u_0) - \Psi _N(t)( u_0)\big ). \end{aligned}$$

By the unitarity of $\Psi _N(0, t)$, (A.29), and the unitarity of $S(-t)$, we have

$$\begin{aligned} \Vert z_N \Vert _{L^2}&= \Vert \Psi (t) (u_0) - \Psi _N(t) (u_0)\Vert _{L^2}\\&= \Vert {\mathcal {G}}\circ \Phi (t) (u_0) - {\mathcal {G}}_N\circ \Phi _N(t) (u_0)\Vert _{L^2}. \end{aligned}$$

By the mean value theorem with (3.2) and (A.28) followed by Lemma 6.20 and the unitarity of $\Phi (t)$, we have

$$\begin{aligned} \Vert z_N \Vert _{L^2}&\le \Vert \Phi (t) (u_0) - \Phi _N(t) (u_0)\Vert _{L^2} + C \big ( \Vert u_0 \Vert _{L^2}^2 - \Vert {\mathbf {P}}_{\le N}u_0 \Vert _{L^2}^2 \big )\Vert \Phi (t) u_0\Vert _{L^2}\nonumber \\&< \frac{\varepsilon }{2} + C R^2 \Vert {\mathbf {P}}_{> N }u_0 \Vert _{L^2} < \varepsilon \end{aligned}$$

for all sufficiently large $N \gg 1$, uniformly in $u_0 \in A \subset B_R$. This proves (A.30). $\square$

## Footnotes

1 In the following, we drop the harmless factor of $2\pi$.

2 Note that (3.6) is non-autonomous. We point out that this non-autonomy does not play an essential role in the remaining part of the paper, since all the estimates hold uniformly in $t \in {\mathbb {R}}$.

3 By viewing $( H^s, H^{s-\sigma _1}, \mu _s)$ as an abstract Wiener space, $DF|_{0}$ is the so-called *H*-derivative of *F* at 0, where $H = H^s$ is the Cameron–Martin space. See [62].

4 By multilinearity, we mean it is either linear or conjugate linear in each argument, i.e. linear over real numbers.

5 This is indeed a normal form reduction applied to the evolution Eq. (6.6) for $\Vert v(t)\Vert _{H^s}^2$. Compare this with the normal form reduction argument in Sect. 5.

6 The normalizing constants $Z_{s, N, r}$ and $Z_{s, r}$ a priori depend on $t \in {\mathbb {R}}$. It is, however, easy to see that they are indeed independent of $t \in {\mathbb {R}}$ by (i) noticing that $R_t(v)$ defined in (6.8) is autonomous in terms of $\widetilde{u} (t)= S(t) v(t)$ and (ii) the invariance of $\mu _s$ under *S*(*t*) (Lemma 4.1).

7 The normalizing constant $\widehat{Z}_{s, N, r}$ a priori depends on $t \in {\mathbb {R}}$. Arguing as for $Z_{s, N, r}$ and $Z_{s, r}$ defined in (6.21) and (6.22), however, we see that it is indeed independent of $t \in {\mathbb {R}}$.

## References

[1] Ablowitz M, Kaup D, Newell D, Segur H (1974). The inverse scattering transform-Fourier analysis for nonlinear problems. Stud. Appl. Math..

[2] Ablowitz M, Ma Y (1981). The periodic cubic Schrödinger equation. Stud. Appl. Math..

[3] Ambrosio L, Figalli A (2009). On flows associated to Sobolev vector fields in Wiener spaces: an approach à la DiPerna–Lions. J. Funct. Anal..

[4] Babin A, Ilyin A, Titi E (2011). On the regularization mechanism for the periodic Korteweg–de Vries equation. Commun. Pure Appl. Math..

[5] Ben-Artzi M, Koch H, Saut JC (2000). Dispersion estimates for fourth order Schrödinger equations. C. R. Acad. Sci. Paris Sér. I Math..

[6] Bogachev, V.: Gaussian measures. In: Mathematical Surveys and Monographs, 62. American Mathematical Society, Providence, pp. xii+433 (1998)

[7] Bourgain J (1993). Fourier transform restriction phenomena for certain lattice subsets and applications to nonlinear evolution equations, I: Schrödinger equations. Geom. Funct. Anal..

[8] Bourgain J (1994). Periodic nonlinear Schrödinger equation and invariant measures. Commun. Math. Phys..

[9] Bourgain J (1996). Invariant measures for the 2D-defocusing nonlinear Schrödinger equation. Commun. Math. Phys..

[10] Bourgain J (1997). Invariant measures for the Gross–Piatevskii equation. J. Math. Pures Appl. (9).

[11] Bourgain J, Bulut A (2014). Invariant Gibbs measure evolution for the radial nonlinear wave equation on the 3d ball. J. Funct. Anal..

[12] Bourgain J, Bulut A (2014). Almost sure global well posedness for the radial nonlinear Schrödinger equation on the unit ball II: the 3D case. J. Eur. Math. Soc. (JEMS).

[13] Burq N, Gérard P, Tzvetkov N (2002). An instability property of the nonlinear Schrödinger equation on $$S^{d}$$. Math. Res. Lett..

[14] Burq N, Thomann L, Tzvetkov N (2013). Long time dynamics for the one dimensional non linear Schrödinger equation. Ann. Inst. Fourier (Grenoble).

[15] Burq, N., Thomann, L., Tzvetkov, N.: Remarks on the Gibbs measures for nonlinear dispersive equations. arXiv:1412.7499 [math.AP]

[16] Burq, N., Tzvetkov, N.: Invariant measure for a three dimensional nonlinear wave equation. Int. Math. Res. Not. IMRN no. 22, Art. ID rnm108, p. 26 (2007)

[17] Burq N, Tzvetkov N (2008). Random data Cauchy theory for supercritical wave equations. I. Local theory. Invent. Math..

[18] Burq, N., Tzvetkov, N.: Random data Cauchy theory for supercritical wave equations. II. A global existence result. Invent. Math. **173**(3), 477–496 (2008)

[19] Cameron R, Martin W (1944). Transformations of Wiener integrals under translations. Ann. Math. (2).

[20] Christ M, Colliander J, Tao T (2003). Asymptotics, frequency modulation, and low regularity ill-posedness for canonical defocusing equations. Am. J. Math..

[21] Christ, M., Colliander, J., Tao, T.: Instability of the periodic nonlinear Schrödinger equation. arXiv:math/0311227v1 [math.AP]

[22] Colliander J, Keel M, Staffilani G, Takaoka H, Tao T (2002). A refined global well-posedness result for Schrödinger equations with derivative. SIAM J. Math. Anal..

[23] Colliander J, Keel M, Staffilani G, Takaoka H, Tao T (2003). Sharp global well-posedness for KdV and modified KdV on $$\mathbb{R}$$ and $$\mathbb{T}$$. J. Am. Math. Soc..

[24] Cruzeiro AB (1983). Équations différentielles ordinaires: non explosion et mesures quasi-invariantes. (Fr.) J. Funct. Anal..

[25] Cruzeiro AB (1983). Équations différentielles sur l’espace de Wiener et formules de Cameron-Martin non-linéaires. (Fr.) J. Funct. Anal..

[26] Da Prato, G.: An introduction to infinite-dimensional analysis. Revised and extended from the 2001 original by Da Prato. Universitext. Springer, Berlin, pp. x+209 (2006)

[27] Deng Y (2012). Two-dimensional nonlinear Schrödinger equation with random radial data. Anal. PDE.

[28] Deng Y (2015). Invariance of the Gibbs measure for the Benjamin–Ono equation. J. Eur. Math. Soc. (JEMS).

[29] Deng Y, Tzvetkov N, Visciglia N (2015). Invariant measures and long time behaviour for the Benjamin–Ono equation III. Commun. Math. Phys..

[30] de Suzzoni A-S (2011). Invariant measure for the cubic wave equation on the unit ball of $$\mathbb{R}^{3}$$. Dyn. Partial Differ. Equ..

[31] de Suzzoni A-S (2014). Wave turbulence for the BBM equation: stability of a Gaussian statistics under the flow of BBM. Commun. Math. Phys..

[32] Erdoğan, M.B., Tzirakis, N.: Global smoothing for the periodic KdV evolution. Int. Math. Res. Not. IMRN (20), 4589–4614 (2013)

[33] Fibich G, Ilan B, Papanicolaou G (2002). Self-focusing with fourth-order dispersion. SIAM J. Appl. Math..

[34] Ginibre J, Tsutsumi Y, Velo G (1997). On the Cauchy problem for the Zakharov system. J. Funct. Anal..

[35] Grébert, B., Kappeler, T.: The defocusing NLS equation and its normal form. EMS Series of Lectures in Mathematics. European Mathematical Society (EMS), Zürich. pp. x+166 (2014)

[36] Gross, L.: Abstract Wiener spaces. Proc. 5th Berkeley Sym. Math. Stat. Prob **2**, 31–42 (1965)

[37] Guo Z, Kwon S, Oh T (2013). Poincaré–Dulac normal form reduction for unconditional well-posedness of the periodic cubic NLS. Commun. Math. Phys..

[38] Guo, Z., Oh, T.: Non-existence of solutions for the periodic cubic nonlinear Schrödinger equation below $$L^2$$. to appear in Internat. Math. Res. Not

[39] Hardy, G.H., Wright, E.M.: An introduction to the theory of numbers, 5th edn. The Clarendon Press, Oxford University Press, New York, pp. xvi+426 (1979)

[40] Ivanov BA, Kosevich AM (1983). Stable three-dimensional small-amplitude soliton in magnetic materials. So. J. Low Temp. Phys..

[41] Karpman VI (1996). Stabilization of soliton instabilities by higher-order dispersion: fourth order nonlinear Schrödinger-type equations. Phys. Rev. E.

[42] Karpman VI, Shagalov AG (1997). Solitons and their stability in high dispersive systems. I. Fourth-order nonlinear Schrödinger-type equations with power-law nonlinearities. Phys. Lett. A.

[43] Kenig C, Ponce G, Gustavo, Vega L (1993). The Cauchy problem for the Korteweg–de Vries equation in Sobolev spaces of negative indices. Duke Math. J..

[44] Koralov L, Sinai Y (2007). Theory of Probability and Random Processes.

[45] Kuo H (1971). Integration theory on infinite-dimensional manifolds. Trans. Am. Math. Soc..

[46] Kuo H (1975). Gaussian measures in Banach spaces. Lecture Notes in Mathematics.

[47] Kwon S, Oh T (2012). On unconditional well-posedness of modified KdV. Int. Math. Res. Not..

[48] Lebowitz J, Rose H, Speer E (1988). Statistical mechanics of the nonlinear Schrödinger equation. J. Stat. Phys..

[49] McKean, H.P.: Statistical mechanics of nonlinear wave equations. IV. Cubic Schrödinger. Commun. Math. Phys. **168**(3), 479–491 (1995) Erratum: Statistical mechanics of nonlinear wave equations. IV. Cubic Schrödinger. Commun. Math. Phys. **173**(3), 675 (1995)

[50] McKean, H.P., Vaninsky, K.L.: Statistical mechanics of nonlinear wave equations. Trends and perspectives in applied mathematics. Appl. Math. Sci., 100, pp. 239–264. Springer, New York (1994)

[51] Molinet L (2009). On ill-posedness for the one-dimensional periodic cubic Schrödinger equation. Math. Res. Lett..

[52] Nahmod A, Oh T, Rey-Bellet L, Staffilani G (2012). Invariant weighted Wiener measures and almost sure global well-posedness for the periodic derivative NLS. J. Eur. Math. Soc..

[53] Nahmod A, Rey-Bellet L, Sheffield S, Staffilani G (2011). Absolute continuity of Brownian bridges under certain gauge transformations. Math. Res. Lett..

[54] Nelson E (1973). The free Markoff field. J. Funct. Anal..

[55] Oh, T.: Invariant Gibbs measures and a.s. global well-posedness for coupled KdV systems. Differ. Integral. Equ. 22 (7–8), 637-668 (2009)

[56] Oh T (2009). Invariance of the white noise for KdV. Commun. Math. Phys..

[57] Oh T (2009). Invariance of the Gibbs Measure for the Schrödinger–Benjamin–Ono system. SIAM J. Math. Anal..

[58] Oh T, Quastel J, Valkó B (2012). Interpolation of Gibbs measures and white noise for Hamiltonian PDE. J. Math. Pures Appl..

[59] Oh, T., Wang, Y.: Global well-posedness of the periodic cubic fourth order NLS in negative Sobolev spaces. preprint

[60] Pausader B (2009). The cubic fourth-order Schrödinger equation. J. Funct. Anal..

[61] Quastel J, Valkó B (2008). KdV preserves white noise. Commun. Math. Phys..

[62] Ramer R (1974). On nonlinear transformations of Gaussian measures. J. Funct. Anal..

[63] Richards, G.: Invariance of the Gibbs measure for the periodic quartic gKdV. to appear in Ann. Inst. H. Poincaré Anal. Non Linéaire

[64] Tao, T.: Nonlinear dispersive equations. Local and global analysis. CBMS Regional Conference Series in Mathematics, 106. Published for the Conference Board of the Mathematical Sciences, Washington, DC; by the American Mathematical Society, Providence, RI, pp. xvi+373 (2006)

[65] Thomann L, Tzvetkov N (2010). Gibbs measure for the periodic derivative nonlinear Schrödinger equation. Nonlinearity.

[66] Turitsyn SK (1985). Three-dimensional dispersion of nonlinearity and stability of multidimensional solitons. Teoret. Mat. Fiz..

[67] Tzvetkov N (2006). Invariant measures for the nonlinear Schrödinger equation on the disc. Dyn. Partial Differ. Equ..

[68] Tzvetkov N (2008). Invariant measures for the defocusing Nonlinear Schrödinger equation (Mesures invariantes pour l’équation de Schrödinger non linéaire). Annales de l’Institut Fourier.

[69] Tzvetkov N (2010). Construction of a Gibbs measure associated to the periodic Benjamin–Ono equation. Probab. Theory Relat. Fields.

[70] Tzvetkov N (2015). Quasi-invariant Gaussian measures for one dimensional Hamiltonian PDE’s. Forum Math. Sigma.

[71] Tzvetkov, N., Visciglia, N.: Invariant measures and long-time behavior for the Benjamin–Ono equation. Int. Math. Res. Not. IMRN (17), 4679–4714 (2014)

[72] Tzvetkov N, Visciglia N (2015). Invariant measures and long time behaviour for the Benjamin–Ono equation II. J. Math. Pures Appl. (9).

[73] Yudovich V (1963). Non-stationary flows of an ideal incompressible fluid. Zh. Vychisl. Math. i Math. Fiz..

[74] Zhidkov P (2001). On an infinite sequence of invariant measures for the cubic nonlinear Schrödinger equation. Int. J. Math. Math. Sci..

[75] Zhidkov P (2001). Korteweg-de Vries and Nonlinear Schrödinger Equations: Qualitative Theory. Lecture Notes in Mathematics.

